# Designing a multi-epitope vaccine against La Crosse virus using immunoinformatics approach

**DOI:** 10.1371/journal.pone.0359567

**Published:** 2026-10-13

**Authors:** Md. Shamiron Hasnat, Ahsanul Siddique Saim, Mst. Farhana Akter Sarmin, Probir Mitro, Sabrina Sarker, Md. Hasan Ali, Shalauddin, Omar Faruk Talukder, Md Bashir Uddin

**Affiliations:** 1 Faculty of Veterinary, Animal and Biomedical Sciences, Sylhet Agricultural University, Sylhet, Bangladesh; 2 Department of Biochemistry and Molecular Biology, Shahjalal University of Science and Technology, Sylhet, Bangladesh; 3 Department of Medicine, Sylhet Agricultural University, Sylhet, Bangladesh; Sefako Makgatho Health Sciences University, SOUTH AFRICA

## Abstract

La Crosse virus (LACV) is a mosquito-borne *Orthobunyavirus* responsible for pediatric encephalitis, with no currently approved vaccine or targeted antiviral therapy. This study aimed to design a multi-epitope subunit vaccine targeting the conserved nucleoprotein of LACV using an integrated immunoinformatics approach. The nucleoprotein exhibited strong antigenicity and favorable physicochemical properties, including stability and hydrophilicity. Highly conserved CTL, HTL, and B-cell epitopes were initially predicted, from which high-affinity, non-allergenic, and non-toxic epitopes were selected. Population coverage analysis revealed extensive global applicability, with MHC class I and II coverage of 98.55% and 81.81%, respectively. Three vaccine constructs (V1, V2 and V3) were developed, while construct V1 demonstrated superior performance, with the highest antigenicity (0.7846) and solubility (0.709). Structural validation confirmed stability, with over 93% of residues in favored regions of the Ramachandran plot. Molecular docking with the TLR8 receptor revealed strong interactions. This was further supported by MD simulations, DCCM analysis, binding free energy calculations, and PCA, all of which confirmed stable binding between the vaccine construct V1 and TLR8, exhibiting a docking score of −1212.6. Molecular dynamics simulations further confirmed stability, with consistent RMSD, stable radius of gyration, and persistent hydrogen bonding (40–60 bonds). Codon optimization demonstrated favorable translational efficiency and confirmed the suitability of the construct V1 for heterologous expression in *E. coli*. Immune simulation predicted robust humoral and cellular responses, including elevated IgM, IgG, and cytokines. Overall, the proposed vaccine candidate V1 demonstrates strong immunogenic potential and structural stability, warranting further experimental validation for potential clinical application.

## Introduction

La Crosse virus (LACV), a mosquito-borne viral infection, member of the genus *Orthobunyavirus* within the family *Peribunyaviridae*, has emerged as a leading cause of pediatric arboviral encephalitis, particularly in North America [1,2]. The virus is primarily transmitted by *Aedes triseriatus* mosquitoes and is maintained in a zoonotic transmission cycle involving small mammals such as chipmunks and squirrels [2]. Human infection occurs incidentally, typically following exposure to infected vectors in forested or peri-urban environments.

Clinically, LACV infection ranges from asymptomatic or mild febrile illness to severe neuroinvasive disease characterized by encephalitis, seizures, and long-term neurological sequelae, particularly in children under 16 years of age [1,3]. Although approximately 80–100 cases are reported annually in the United States, the actual disease burden is likely underestimated due to subclinical infections and diagnostic limitations [2]. Despite its clinical significance, no licensed vaccines or targeted antiviral therapies are currently available, and treatment remains largely supportive.

The LACV genome comprises three negative-sense RNA segments (L, M, and S), encoding essential structural and non-structural proteins involved in viral replication and host interaction [3]. Among these, the nucleoprotein plays a critical role in viral RNA encapsidation and is relatively conserved across strains, making it an attractive target for vaccine development. Targeting conserved viral proteins reduces the risk of antigenic escape and enhances the potential for broad-spectrum protection [4,5].

Traditional vaccine development approaches, including live attenuated and inactivated vaccines, are often time-consuming and may present safety concerns, particularly for neurotropic viruses. Recent advances in computational biology have facilitated the emergence of immunoinformatics and reverse vaccinology approaches, enabling rapid identification of potential vaccine candidates directly from genomic data [6]. These approaches allow systematic screening of viral proteins to identify immunogenic epitopes capable of eliciting robust immune responses while minimizing adverse effects.

Epitope-based vaccine design has gained considerable attention as a next-generation strategy for vaccine development. Unlike conventional vaccines, multi-epitope subunit vaccines incorporate selected B-cell and T-cell epitopes to stimulate both humoral and cellular immunity [7,8]. Cytotoxic T lymphocyte (CTL) epitopes activate CD8 ⁺ T cells, helper T lymphocyte (HTL) epitopes stimulate CD4 ⁺ T cells, and B-cell epitopes promote antibody production. The integration of these epitopes into a single construct enhances immunogenicity and ensures comprehensive immune activation [9,10].

Furthermore, structural bioinformatics and molecular modeling tools enable prediction of protein structure, validation of stability, and evaluation of interactions with immune receptors such as Toll-like receptors (TLRs), which play a key role in innate immune activation [11]. Molecular docking and immune simulation analyses further facilitate the assessment of vaccine efficacy prior to experimental validation.

Given the increasing geographic spread of mosquito vectors due to climate change and environmental factors, the development of effective vaccines against LACV is urgently needed. Therefore, this study aims to design a multi-epitope subunit vaccine targeting the conserved nucleoprotein of LACV using an integrated immunoinformatics approach. The study incorporates epitope prediction, structural modeling, molecular docking, immune simulation, and *in silico* cloning to evaluate the immunogenicity, stability, and feasibility of the proposed vaccine construct.

## Methodology

A reverse-vaccinology and immunoinformatics workflow was implemented to identify, prioritize, and validate candidate epitopes for a multi-epitope vaccine against La Crosse virus (LACV). The diagram integrated antigen retrieval, physicochemical and topological characterization, B- and T-cell epitope prediction, construct assembly, structural modeling, receptor docking, molecular dynamics analysis, immune simulation, and in silico cloning. The complete workflow is summarized in Fig 1.

**Fig 1:**
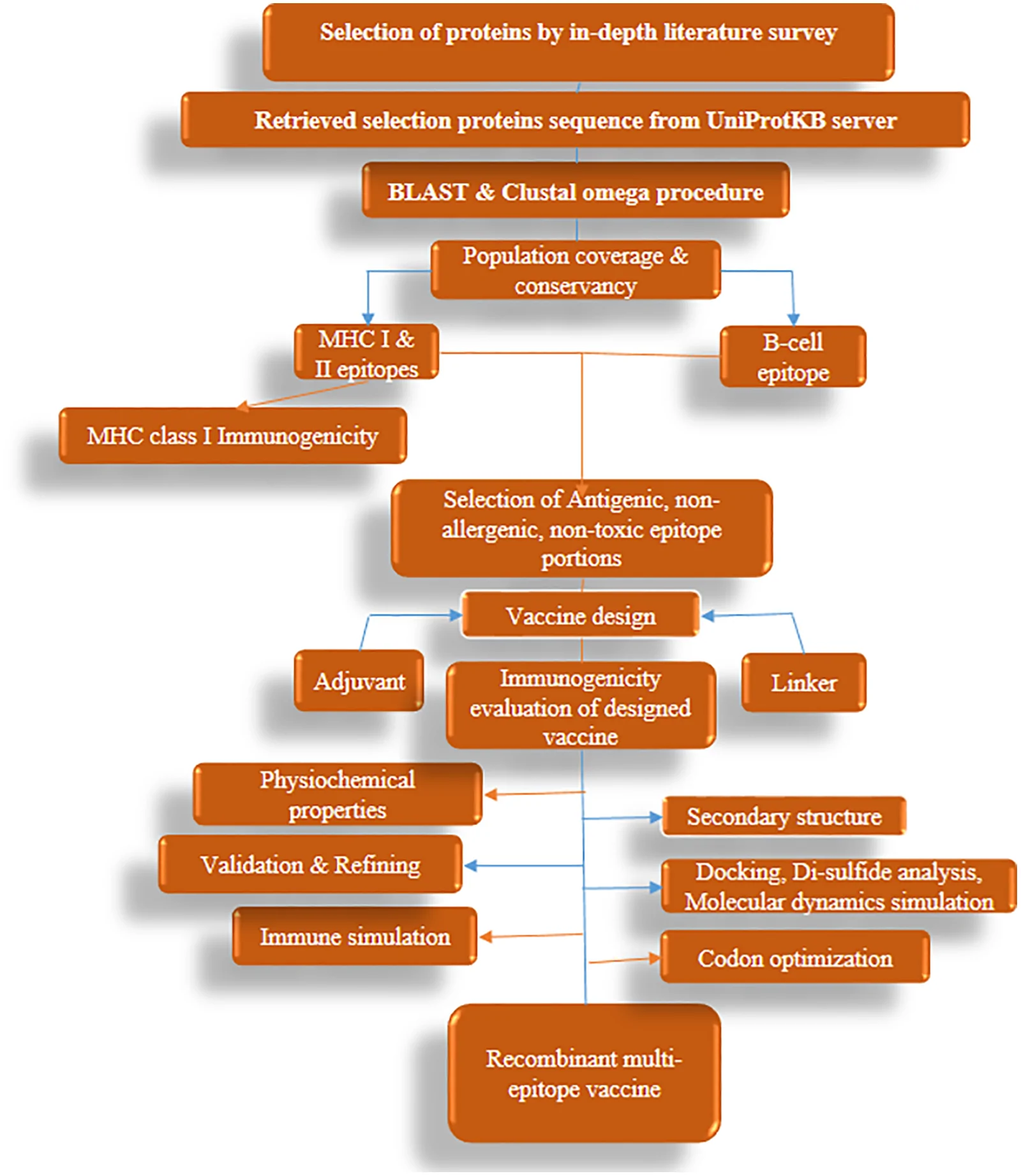
An outline of the steps involved in employing an in silico reverse vaccinology approach to predict epitope-based vaccination candidates.

### Sequence retrieval and antigenicity assessment

The amino acid sequence of the LACV nucleoprotein (UniProtKB accession ID: P04873) was obtained from the UniProtKB database and used as the primary antigenic target. This sequence corresponds to the reference nucleoprotein of La Crosse virus isolate L78, originally isolated in the United States in 1960. The isolate belongs to La Crosse virus (California serogroup; genus *Orthobunyavirus*; family *Peribunyaviridae*). A specific phylogenetic lineage has not been assigned to this reference isolate in the UniProtKB record. The sequence was selected because it is a UniProtKB/Swiss-Prot protein accession corresponding to the nucleoprotein (N protein) of LACV with complete functional annotation, high sequence quality, and broad acceptance as a reference sequence for LACV studies. Furthermore, the nucleoprotein is highly conserved among LACV strains and plays an essential role in viral RNA encapsidation and replication, making it an attractive target for epitope-based vaccine design. Antigenicity was assessed using the VaxiJen v2.0 server, which classifies proteins according to alignment-independent physicochemical descriptors [12,13].

The biochemical profile of the target protein was characterized with ProtParam, including molecular weight, theoretical isoelectric point (pI), instability index, aliphatic index, and grand average of hydropathicity (GRAVY). These parameters were used to infer protein stability, hydrophilicity, and formulation suitability. Transmembrane architecture was predicted with TMHMM v2.0 to distinguish exposed regions with potential accessibility to host immune recognition [14].

### Conserved region identification and B-cell epitope prediction

NCBI protein BLAST was used to look for homologous sequences in La Crosse virus (taxid:3052409), as this serotype is the most commonly associated with human infection cases. The resulting homologous sequences were screened based on sequence similarity and statistical significance, and sequences showing E-values close to zero were selected. A total of 13 representative nucleoprotein sequences (S1 Table) were included for multiple sequence alignment using Clustal Omega. These sequences were selected based on high similarity, low E-values, and availability of complete and annotated nucleoprotein sequences. ClustalOmega server was then applied to specify the conserved regions among the homologous sequences for nucleoprotein followed by the use of Vaxijen server and TMHMM server to reveal the antigenicity and topology of the conserved region and aligned with Clustal Omega to define conserved antigenic segments. Conservation screening was included to prioritize epitopes less likely to be affected by strain-level variation and to support broader protective coverage [15]. Linear B-cell epitopes were predicted through the Immune Epitope Database (IEDB) using BepiPred 2.0, Emini surface accessibility, and Kolaskar–Tongaonkar antigenicity approaches [16,17]. Together, these tools enabled the identification of peptide regions predicted to be antigenic, solvent-accessible, and suitable for humoral immune recognition.

Predicted B-cell epitopes were subsequently filtered for antigenicity using VaxiJen, allergenicity using AllerTOP v2.0 and AllerCatPro, and toxicity using established in silico screening criteria. Only epitopes with favorable antigenic profiles, non-allergenic predictions, and non-toxic properties were retained for downstream vaccine design, consistent with multi-parameter epitope selection strategies used in contemporary immunoinformatics pipelines [18,19].

### Prediction of T-cell epitopes (HTL and CTL)

Helper T lymphocyte (HTL) epitopes were predicted using the IEDB MHC class II binding prediction platform [20]. Candidate peptides were ranked on the basis of binding affinity, with priority given to epitopes showing strong binding potential (IC_50_ < 500 nM) and favorable percentile ranks. Cytotoxic T lymphocyte (CTL) epitopes were identified using IEDB MHC class I binding tools and were similarly prioritized according to predicted interaction strength with representative HLA alleles.

Both HTL and CTL candidates were subjected to sequential screening for antigenicity, allergenicity, toxicity, and extracellular accessibility. This combined selection strategy was applied to retain epitopes capable of supporting coordinated humoral and cellular responses, which are essential for antiviral protection (Nguyen et al., 2025). The inclusion of both CTL and HTL epitopes was intended to promote CD8 ⁺ T-cell cytotoxicity together with CD4 ⁺ T-cell assistance, thereby improving the immunological breadth of the vaccine construct [10,15].

### Population coverage and epitope conservancy analysis

The IEDB population coverage tool was used to estimate the distribution of HLA alleles associated with the selected epitopes across global and regional populations. This analysis was performed to evaluate the potential applicability of the epitope set across genetically diverse human groups. Epitope conservancy was also assessed to determine the degree of sequence preservation among LACV strains; highly conserved epitopes were prioritized to strengthen the likelihood of cross-protective immunity. The regions included in the population coverage analysis were selected based on the known geographical distribution of LACV, the availability of HLA allele frequency data in the IEDB Population Coverage tool, and the relevance of these regions to the epidemiology of the virus. North America, particularly the United States where LACV is endemic, was prioritized because it represents the primary area of virus circulation. Additional regions were included where comprehensive HLA frequency data were available to evaluate the potential global applicability of the predicted vaccine candidates. Regions lacking sufficient HLA allele frequency data or with limited epidemiological relevance to LACV transmission were not included in the analysis. In addition, MHCcluster v2.0 was used to examine functional similarity and clustering patterns among selected MHC alleles, providing further support for the expected breadth of peptide presentation [21].

### Multi-epitope vaccine construction

Three multi-epitope vaccine constructs, designated V1, V2, and V3, were generated by assembling the prioritized CTL, HTL, and B-cell epitopes with appropriate adjuvants and a Pan HLA-DR-binding Epitope (PADRE) sequence. Adjuvant sequences were included to strengthen innate immune stimulation and enhance antigen presentation, whereas PADRE was incorporated to improve helper T-cell responsiveness. Linkers were selected to maintain separation, flexibility, and independent epitope presentation within the constructs. The EAAAK linker was placed between adjuvant and epitope modules, whereas GGGS, GPGPG, and KK linkers were used to join CTL, HTL, and B-cell epitope regions, respectively. This linker strategy was applied to minimize steric interference and preserve epitope functionality [22,23].

### Structural modeling and validation

The designed vaccine constructs were evaluated for antigenicity, allergenicity, and solubility using VaxiJen, AllerTOP, AllerCatPro, and Protein-sol servers. Secondary structural features, including α-helices, β-sheets, and coil regions, were predicted using PSIPRED. Three-dimensional models were generated using GalaxyWEB and subsequently refined with GalaxyRefine to improve model quality. Structural validity was examined using PROCHECK-based Ramachandran plot assessment and ERRAT analysis, enabling evaluation of stereochemical reliability and overall model quality [24].

### Molecular docking analysis

Protein–protein docking was performed to characterize interactions between the vaccine constructs and immune receptors, including Toll-like receptor 8 (TLR-8) and selected major histocompatibility complex (MHC) molecules. ClusPro 2.0 was used to generate docked complexes, rank binding orientations, and estimate interaction energies [25]. The docking outputs were used to identify the construct with the most favorable receptor association for subsequent dynamic and energetic analyses.

### Molecular dynamics simulation

The molecular dynamics (MD) simulation of the vaccine–immune receptor complex was performed using GROMACS with the CHARMM36 force field. The protein–protein complex was prepared using the CHARMM36 force field parameters, and the system was solvated in a suitable water model. Counter ions were added to neutralize the system, followed by energy minimization to remove steric clashes and unfavorable interactions. After energy minimization, the system was subjected to equilibration under NVT and NPT ensembles. The temperature was maintained at 310 K, and the pressure was maintained at 1 bar using appropriate coupling algorithms. A production MD simulation was subsequently performed for 200 ns under periodic boundary conditions. The stability and dynamic behavior of the complex were evaluated through several trajectory analyses, including root mean square deviation (RMSD), root mean square fluctuation (RMSF), radius of gyration (Rg), solvent accessible surface area (SASA), hydrogen bond analysis, principal component analysis (PCA), and residue cross-correlation analysis.

### Immune simulation and in silico cloning

The immunogenic profile of the selected vaccine construct was assessed using the C-ImmSim server to predict humoral and cellular immune responses, including antibody kinetics, cytokine secretion, and memory cell development. Codon optimization was performed using JCAT to improve heterologous expression potential in Escherichia coli. The optimized coding sequence was then inserted into a pET expression vector using SnapGene. Codon adaptation index (CAI) and GC content were evaluated to determine the suitability of the optimized construct for transcriptional and translational efficiency.

## Results

### Selection and characterization of target protein

The LACV nucleoprotein was selected as the vaccine target because of its conserved nature and functional role in viral RNA encapsidation and replication. The retrieved UniProtKB sequence (Accession ID: P04873) produced a VaxiJen score above the antigenicity threshold (>0.4), supporting its potential to elicit an immune response. ProtParam analysis further indicated a favorable physicochemical profile, including an instability index below 40, a moderate aliphatic index, and a negative GRAVY value (S2 Table). These characteristics suggest that the nucleoprotein is stable and hydrophilic, making it appropriate for subsequent epitope prediction and construct development.

### Conserved regions and B-cell epitopes

Multiple sequence alignment of homologous nucleoprotein sequences revealed five conserved regions among the analyzed LACV strains. Of these, three regions showed strong antigenic potential and were advanced for epitope-level screening. IEDB-based B-cell epitope prediction identified several candidate linear epitopes, which were refined using antigenicity, surface accessibility, allergenicity, and toxicity criteria. Four high-confidence B-cell epitopes fulfilled the selection requirements (S3 Table), indicating their suitability for inclusion in a vaccine construct designed to promote antibody-mediated immune responses.

### Prioritization of T-cell epitopes

Initial MHC-binding prediction generated 1053 HTL (MHC-II) and 2997 CTL (MHC-I) candidate epitopes (S1 & S2 File). These candidates were progressively filtered according to predicted binding affinity, IC_50_ values, antigenicity, allergenicity, and toxicity. The retained HTL epitopes exhibited strong MHC class II binding profiles, including IC_50_ values below 500 nM and favorable percentile ranks, whereas the selected CTL epitopes showed high-affinity binding to MHC class I alleles with predicted immunogenic potential. All selected epitopes were also predicted to be extracellular, supporting their accessibility to immune surveillance (S4 & S5 Table). The final T-cell epitope panel therefore combined safety, antigenicity, and binding strength suitable for multi-epitope vaccine assembly.

### Population coverage and epitope conservancy analysis

The selected epitopes were evaluated for global and regional HLA population coverage using the IEDB population coverage tool (Fig 2). MHC class I-restricted epitopes achieved a predicted global coverage of 98.55%, with an average hit of 6.9. Region-specific coverage was also high, including 99.1% in the United States, 88.3% in South America, 94.51% in Australia, 99.27% in Russia, 99.7% in Central Africa, 99.68% in Europe, 98.18% in East Asia, and 99.06% in North America. For MHC class II-restricted epitopes, the estimated global coverage was 81.81%, with an average hit of 4.43. Regional MHC class II coverage was 88.1% in the United States, 58.59% in South America, 33.15% in Australia, 62.71% in Central Africa, 85.83% in Europe, 81.82% in East Asia, 77.62% in Russia, and 87.89% in North America. Although MHC class II coverage was lower than MHC class I coverage, the combined dataset indicates broad applicability across diverse populations. MHC cluster analysis demonstrated the functional relationships among the selected MHC class I and class II alleles (Fig 3). The radial phylogenetic tree of the MHC class I alleles (Fig 3A) grouped alleles with similar peptide-binding characteristics into closely related clusters, while the corresponding hierarchical heatmap (Fig 3B) revealed strong functional similarities among several HLA class I molecules, as indicated by the prominent red clusters. Similarly, the phylogenetic tree of the MHC class II alleles (Fig 3C) and the associated heatmap (Fig 3D) showed clustering of functionally related HLA class II molecules based on their peptide-binding specificity. Overall, these results suggest that the selected epitopes are capable of interacting with multiple functionally related MHC class I and class II alleles, thereby supporting broad antigen presentation and complementing the high population coverage predicted by the IEDB analysis.

**Fig 2:**
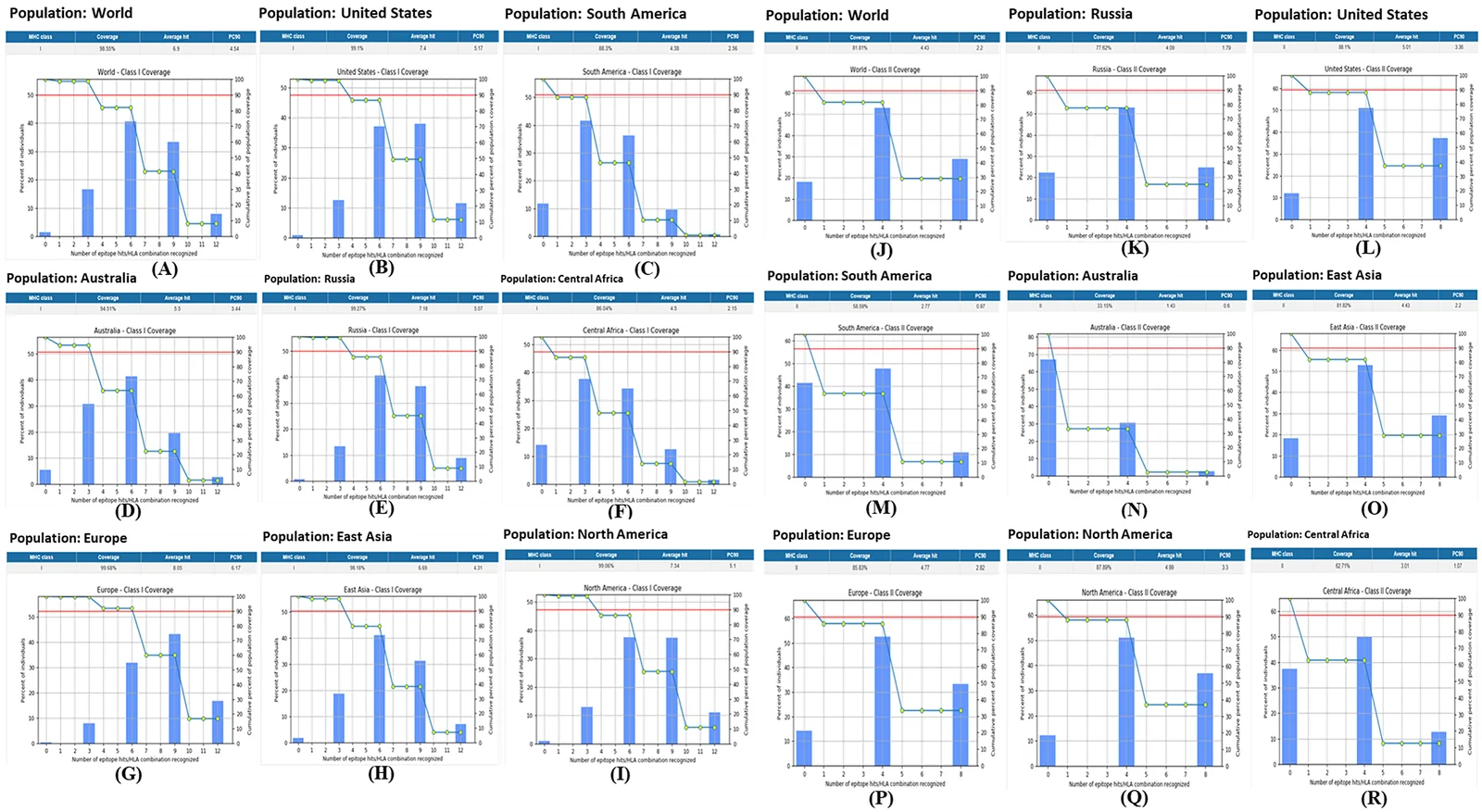
Population coverage of selected (i) MHC I epitopes and (ii) MHC II epitopes.

**Fig 3:**
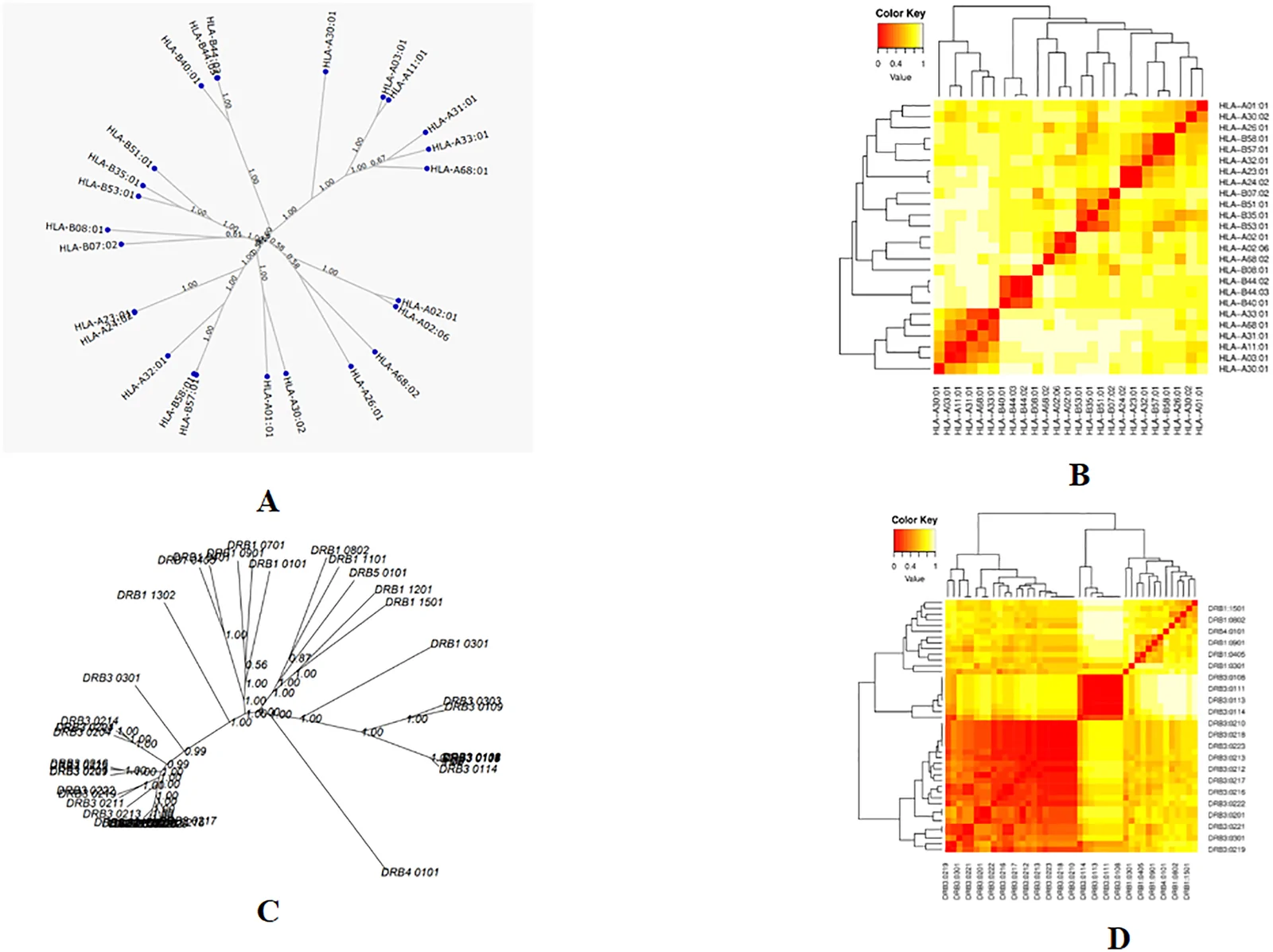
Functional clustering analysis of selected HLA class I and class II alleles. (A) Radial phylogenetic tree illustrating the functional relationships among the selected HLA class I alleles based on predicted peptide-binding specificities. (B) Hierarchical clustering heatmap of HLA class I alleles showing pairwise functional similarity. (C) Radial phylogenetic tree illustrating the functional relationships among the selected HLA class II alleles. (D) Hierarchical clustering heatmap of HLA class II alleles based on peptide-binding similarity. In the heatmaps, red indicates the highest functional similarity between HLA alleles, orange represents high similarity, yellow indicates moderate similarity, and white denotes low or negligible similarity.

Conservancy assessment showed that several selected epitopes were fully conserved across the analyzed viral strains. This complete conservation supports the potential for cross-strain protection and lowers the likelihood of immune escape caused by antigenic variation. MHC cluster analysis further demonstrated functional similarity among multiple HLA alleles, supporting the predicted breadth of epitope presentation.

### Construction of vaccine candidates

Three vaccine constructs (V1, V2, and V3) were developed by combining selected CTL, HTL, and B-cell epitopes with distinct protein adjuvants and PADRE sequences. The adjuvant region was separated from the epitope cassette using an EAAAK linker. Each construct incorporated three CTL epitopes, four HTL epitopes, and four B-cell epitopes arranged with linkers selected to preserve flexibility and epitope exposure. GGGS linkers connected CTL epitopes and the PADRE region, GPGPG linkers joined HTL epitopes and separated CTL and HTL segments, and KK linkers connected HTL and B-cell regions as well as adjacent B-cell epitopes. The final B-cell epitope was linked to the PADRE sequence with KK linkers, and the construct was terminated with a GGGS linker (Fig 4 and Table 1).

**Table 1:** Assessment of the developed vaccines allergenicity, solubility, and antigenicity.

	Composition	Complete Sequence of Vaccine Constructs	No. of residues	Allergenicity	Solubility	VaxiJen score (Threshold 0.4)
V1	Predicted CTL, HTL & BCL epitopes of LACV nucleoprotein with β defensin adjuvant & PADRE sequence	**EAAAK**GIINTLQKYYCRVRGGRCAVLSCLPKEEQIGKCSTRGRKCCRRKK**EAAAK**AKFVAAWTLKAAA**GGGSKFYPLTIGIHGGGSASTGANGFDPGGGSYLSFFPGTEGPGPGTFKFYPLTIGIHRVKGPGPGFKFYPLTIGIHRVKQGPGPGKFYPLTIGIHRVKQGGPGPGLETFKFYPLTIGIHRKK**SLNLAAV**KK**QGMMDPQY**KK**DAGYMD**KK**AESLNLA**KK**AKFVAAWTLKAAA**GGGS**	244	Non-Allergen	0.709	**0.7846**
V2	Predicted CTL, HTL & BCL epitopes of LACV nucleoprotein with L7/ L12 ribosomal protein adjuvant & PADRE sequence	**EAAAK**MAKLSTDELLDAFKEMTLLELSDFVKKFEETFEVTAAAPVAVAAAGAAPAGAAVEAAEEQSEFDVILEAAGDKKIGVIKVVREIVSGLGLKEAKDLVDGAPKPLLEKVAKEAADEAKAKLEAAGATVTVK**EAAAK**AKFVAAWTLKAAA**GGGSKFYPLTIGIHGGGSASTGANGFDPGGGSYLSFFPGTEGPGPGTFKFYPLTIGIHRVKGPGPGFKFYPLTIGIHRVKQGPGPGKFYPLTIGIHRVKQGGPGPGLETFKFYPLTIGIHRKK**SLNLAAV**KK**QGMMDPQY**KK**DAGYMD**KK**AESLNLA**KK**AKFVAAWTLKAAA**GGGS**	329	Non-Allergen	0.530	**0.6645**
V3	Predicted CTL, HTL & BCL epitopes of LACV nucleoprotein with HABA adjuvant & PADRE sequence	**EAAAK**MAENPNIDDLPAPLLAALGAADLALATVNDLIANLRERAEETRAETRTRVEERRARLTKFQEDLPEQFIELRDKFTTEELRKAAEGYLEAATNRYNELVERGEAALQRLRSQTAFEDASARAEGYVDQAVELTQEALGTVASQTRAVGERAAKLVGIEL**EAAAK**AKFVAAWTLKAAA**GGGSKFYPLTIGIHGGGSASTGANGFDPGGGSYLSFFPGTEGPGPGTFKFYPLTIGIHRVKGPGPGFKFYPLTIGIHRVKQGPGPGKFYPLTIGIHRVKQGGPGPGLETFKFYPLTIGIHRKK**SLNLAAV**KK**QGMMDPQY**KK**DAGYMD**KK**AESLNLA**KK**AKFVAAWTLKAAA**GGGS**	358	Non-Allergen	0.541	**0.6885**

**Fig 4:**
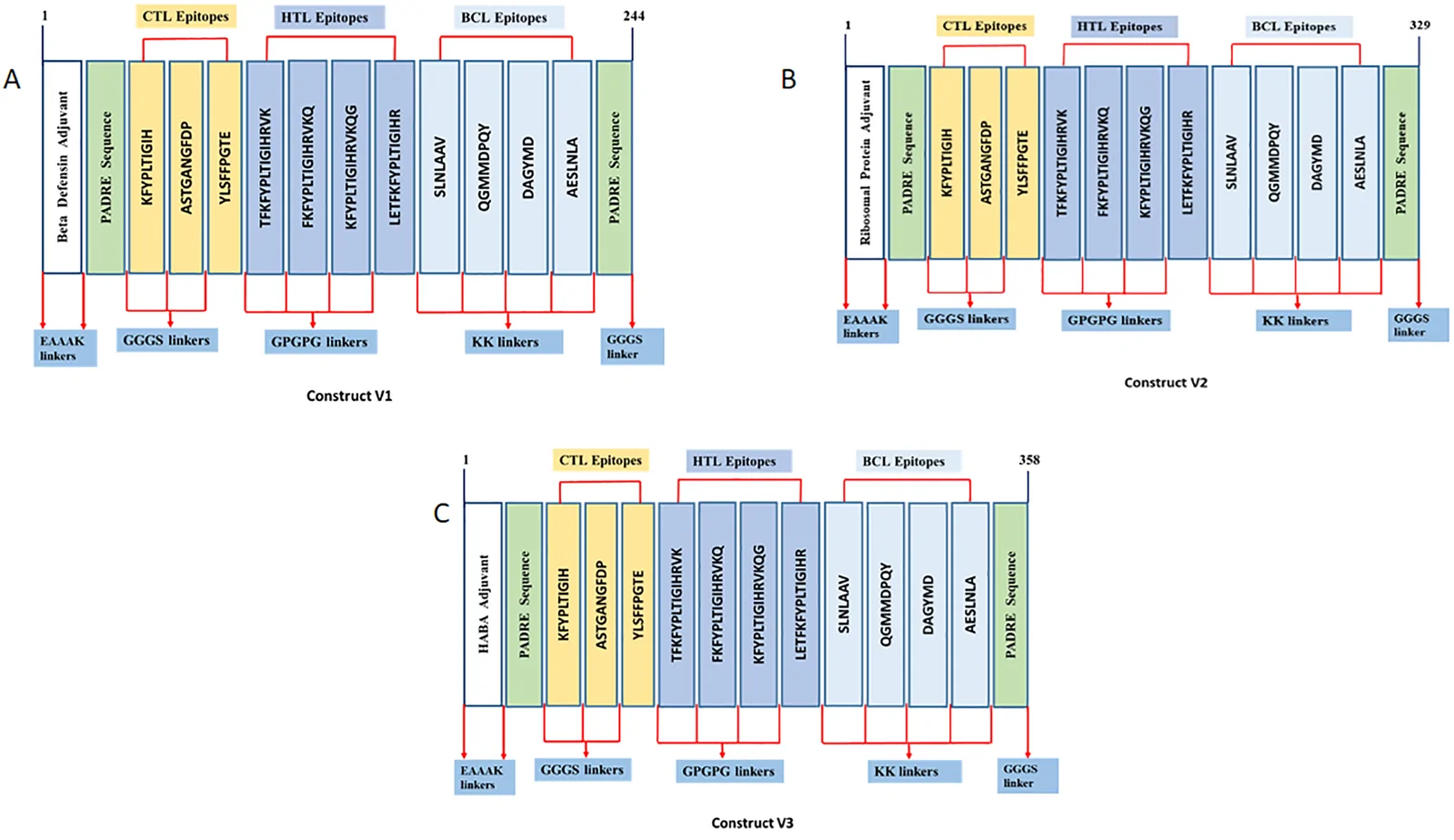
Schematic representation of the multi-epitope vaccine constructs V1, V2, and V3. The 244-, 329-, and 358-amino-acid constructs contain an adjuvant, PADRE sequence, CTL epitopes, HTL epitopes, and B-cell epitopes joined using EAAAK, GGGS, GPGPG, and KK linkers.

The three vaccine constructs showed antigenicity scores above the VaxiJen threshold. V1 had the highest predicted antigenicity score (0.7846), followed by V3 (0.6885) and V2 (0.6645). Protein-sol analysis predicted solubility scores of 0.709 for V1, 0.530 for V2, and 0.541 for V3 (Fig 5), all exceeding the standard solubility threshold of 0.45. These results indicate that all constructs were soluble, with V1 showing the most favorable combined antigenicity and solubility profile (Table 1).

**Fig 5:**
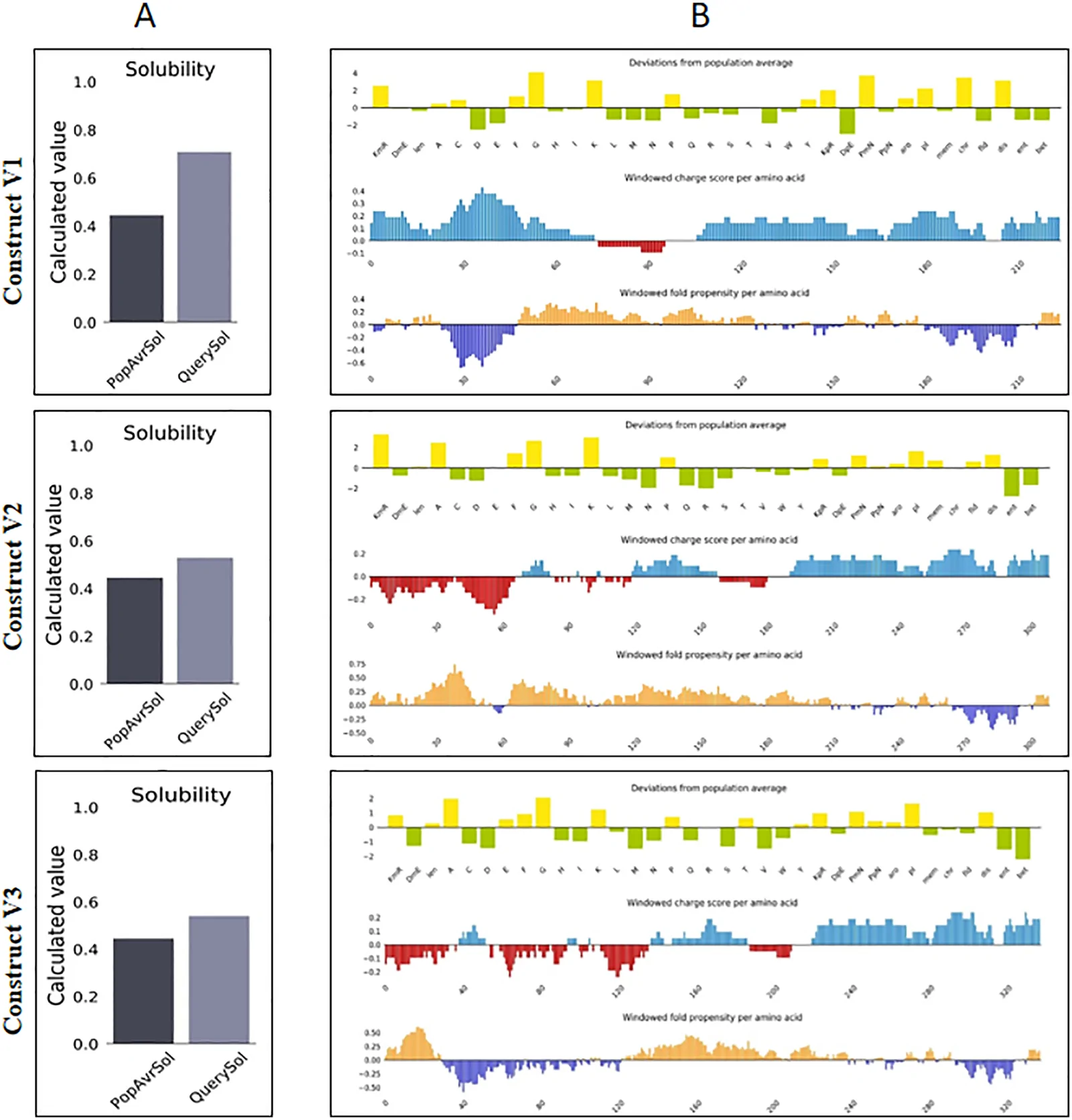
Protein-sol-based solubility prediction for vaccine constructs V1, V2, and V3. (A) Solubility comparison between the population average and each designed construct. (B) Residue-wise profiles showing fold propensity, windowed charge score, and deviation from the population average.

### Physicochemical evaluation of multi-epitope vaccine candidates

Physicochemical analysis was performed to evaluate the biochemical stability and structural suitability of V1, V2, and V3 (Table 2). The constructs contained 244, 329, and 358 amino acids, respectively, with predicted molecular weights of 25,827.20 Da, 34,106.49 Da, and 38,294.65 Da. The theoretical pI values were 10.14 for V1, 9.38 for V2, and 9.41 for V3, indicating that all constructs are basic proteins. Total atom counts were 3,677 for V1, 4,879 for V2, and 5,427 for V3, whereas the predicted extinction coefficients were 26,275, 22,920, and 27,390, respectively.

**Table 2:** Physicochemical characteristics of the vaccine constructs (V1, V2 and V3).

Physicochemical properties	V1	V2	V3
Number of amino acids	244	329	358
Molecular weight	25827.20	34106.49	38294.65
Theoretical pI	10.14	9.38	9.41
Formula	C_1174_H_1855_N_329_O_310_S_9_	C_1559_H_2469_N_403_O_443_S_5_	C_1717_H_2721_N_481_O_504_S_4_
Total number of atoms	3677	4879	5427
Extinction coefficients	26275	22920	27390
Estimated half-life	>10 hours	>10 hours	>10 hours
Instability index	33.04	26.36	36.41
Aliphatic index	66.52	81.16	77.35
Grand average of hydropathicity (GRAVY)	−0.305	−0.059	−0.340

All constructs had an estimated half-life greater than 10 hours. Their instability indices—33.04 for V1, 26.36 for V2, and 36.41 for V3—were below the stability threshold of 40, indicating stable protein behavior. Aliphatic index values of 66.52, 81.16, and 77.35 for V1, V2, and V3, respectively, suggest favorable thermostability. Negative GRAVY values for V1 (−0.305), V2 (−0.059), and V3 (−0.340) further indicate hydrophilic properties, supporting aqueous compatibility and potential suitability for recombinant vaccine development.

### Secondary and tertiary structure modeling and validation

PSIPRED analysis showed that the vaccine constructs contained α-helical, β-sheet, and coil elements, indicating an organized secondary structural composition (S1 Fig in S4 File). Three-dimensional models generated and refined through GalaxyWEB/GalaxyRefine showed improved structural features after refinement. This visualization facilitates the assessment of the overall folding pattern and structural organization of the vaccine constructs (Fig 6). Ramachandran plot validation placed more than 92–93% of residues in favored regions across the constructs, indicating acceptable stereochemical quality (Fig 7 & S2 Fig in S4 File). ERRAT analysis further supported the reliability of the predicted models. These findings suggest that the constructs, particularly V1, maintained stable conformations appropriate for receptor interaction and immune recognition.

**Fig 6:**
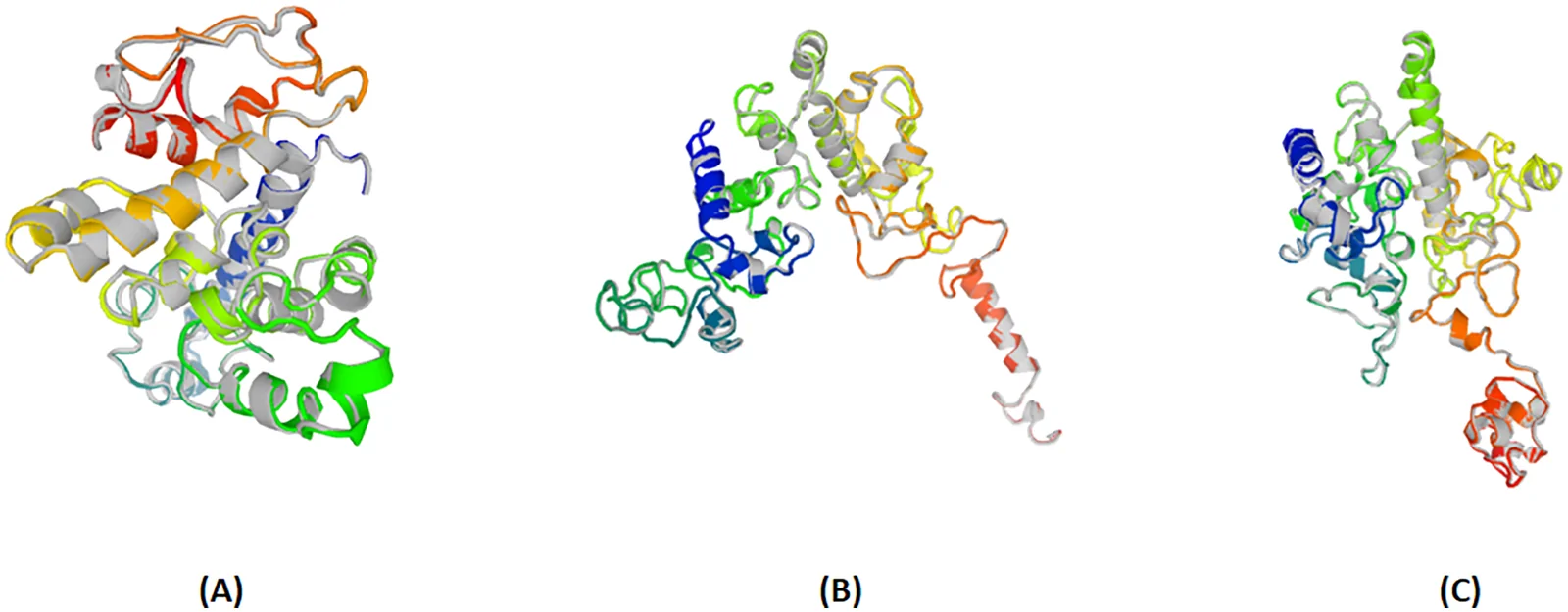
Refined three-dimensional structures of the designed vaccine constructs: (A) Vaccine construct V1. (B) Vaccine construct V2. (C) Vaccine construct V3. The structures are displayed in a rainbow color gradient, where blue represents the N-terminal region, followed sequentially by cyan, green, yellow, and orange, while **red represents the C-terminal region**. This color transition illustrates the residue order along the protein chain and facilitates visualization of the overall folding pattern and structural organization of each vaccine construct.

**Fig 7:**
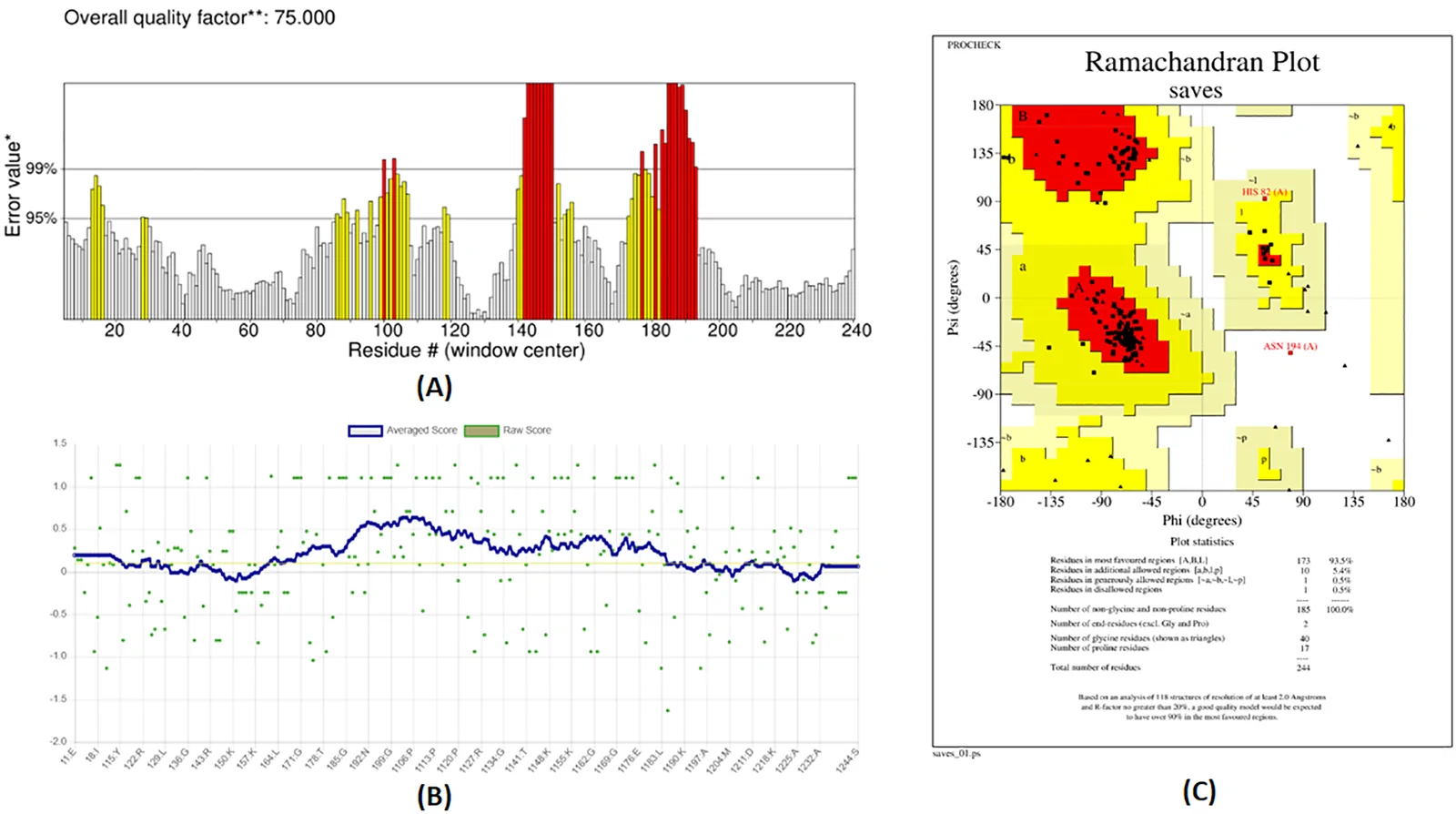
Tertiary structure prediction and validation of vaccine construct V1. (A) ERRAT-based model quality assessment. (B) Verify3D profile of the selected model, with blue indicating the average score. (C) Ramachandran plot analysis.

### Molecular docking analysis with immune receptors

Docking analysis indicated that the vaccine constructs interacted favorably with immune receptors, with the strongest interaction observed for V1 against Toll-like receptor 8 (TLR-8). V1 produced the lowest docking energy score (−1212.6), indicating formation of a highly stable vaccine–TLR8 complex (Fig 8). The construct also showed favorable docking with multiple MHC class I and class II molecules, supporting its potential for antigen presentation (Table 3). Because TLR-8 is involved in viral RNA sensing and innate immune activation, the strong V1–TLR8 interaction suggests that the construct may efficiently engage early antiviral immune pathways.

**Table 3:** Predicted epitopes’ binding energies with certain MHC class I and II molecules as determined by molecular docking.

HLA alleles/ TLRs PDB ID’s	ClusPro 2.0 server	Vaccine Construct		
**Energy score**	**V1**	**V2**	**V3**
**1A6A**	Center	−862.5	−745.8	−865.8
	Lowest	−971.0	−1003.7	−918.8
**2SEB**	Center	−835.6	−828.4	−936.7
	Lowest	−934.1	−1044.5	−1051.4
**2Q6W**	Center	−931.1	−945.2	−872.3
	Lowest	−1136.7	−1039.7	−917.0
**2FSE**	Center	−1007.1	−856.8	−782.5
	Lowest	−1119.7	−1021.4	−1066.8
**1H15**	Center	−951.6	−878.5	−720.3
	Lowest	−1007.1	−966.1	−855.0
TLR-8 (3W3M)	Center	−829.7	−900.8	−849.5
	Lowest	−1212.6	−954.4	−1150.7

**Fig 8:**
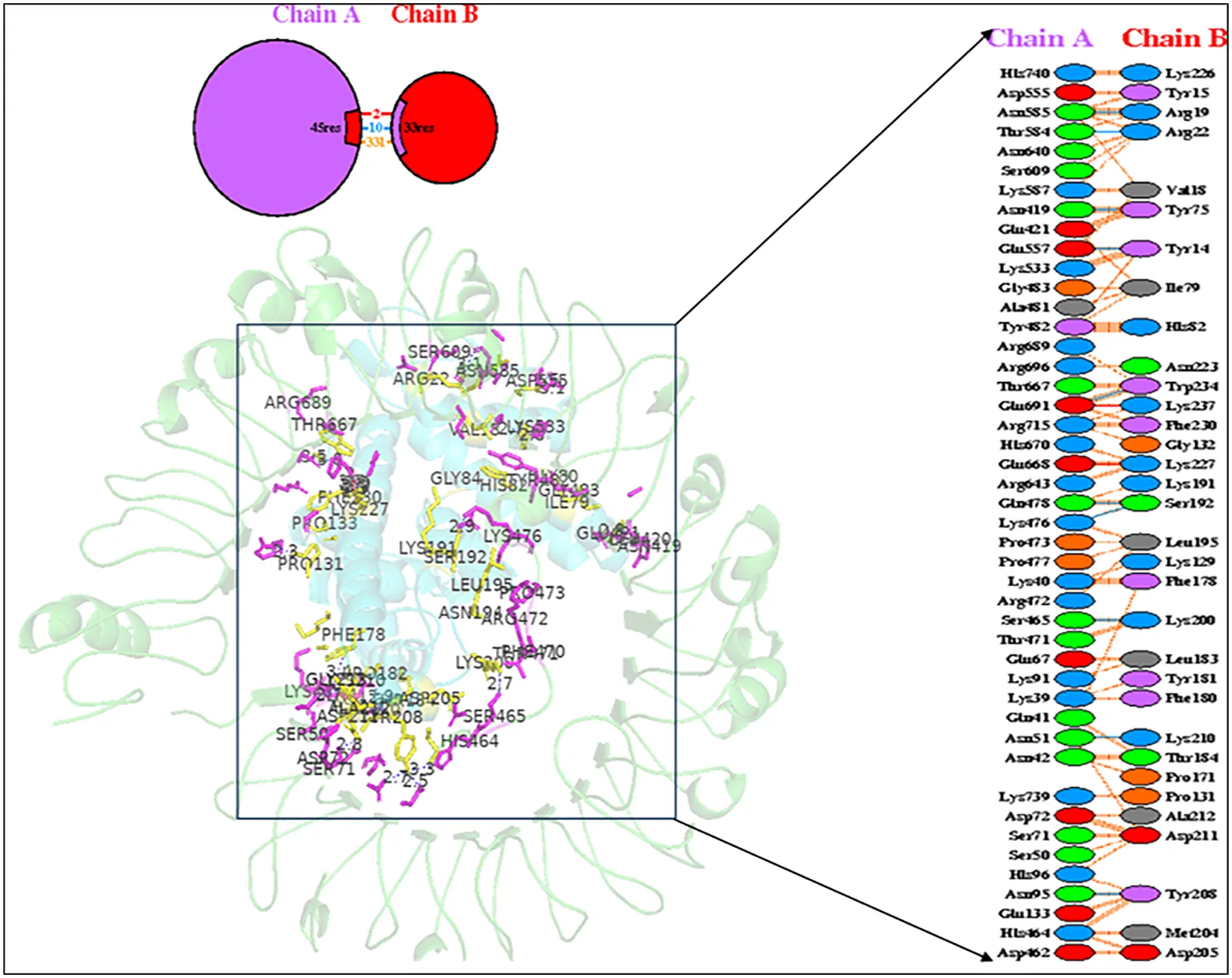
Molecular docking interaction of multi-epitope vaccine construct V1 with the TLR8 receptor, showing the binding interface and residue-level interactions.

Ribbon and atomic representations further indicated compact folding and appropriate spatial organization of V1 (Fig 9). The V1-derived docking models were positioned within the TLR8 interaction region (S3 Fig in S4 File). Among these, models D and E displayed broader interface coverage, suggesting more extensive receptor association and potentially improved binding stability.

**Fig 9:**
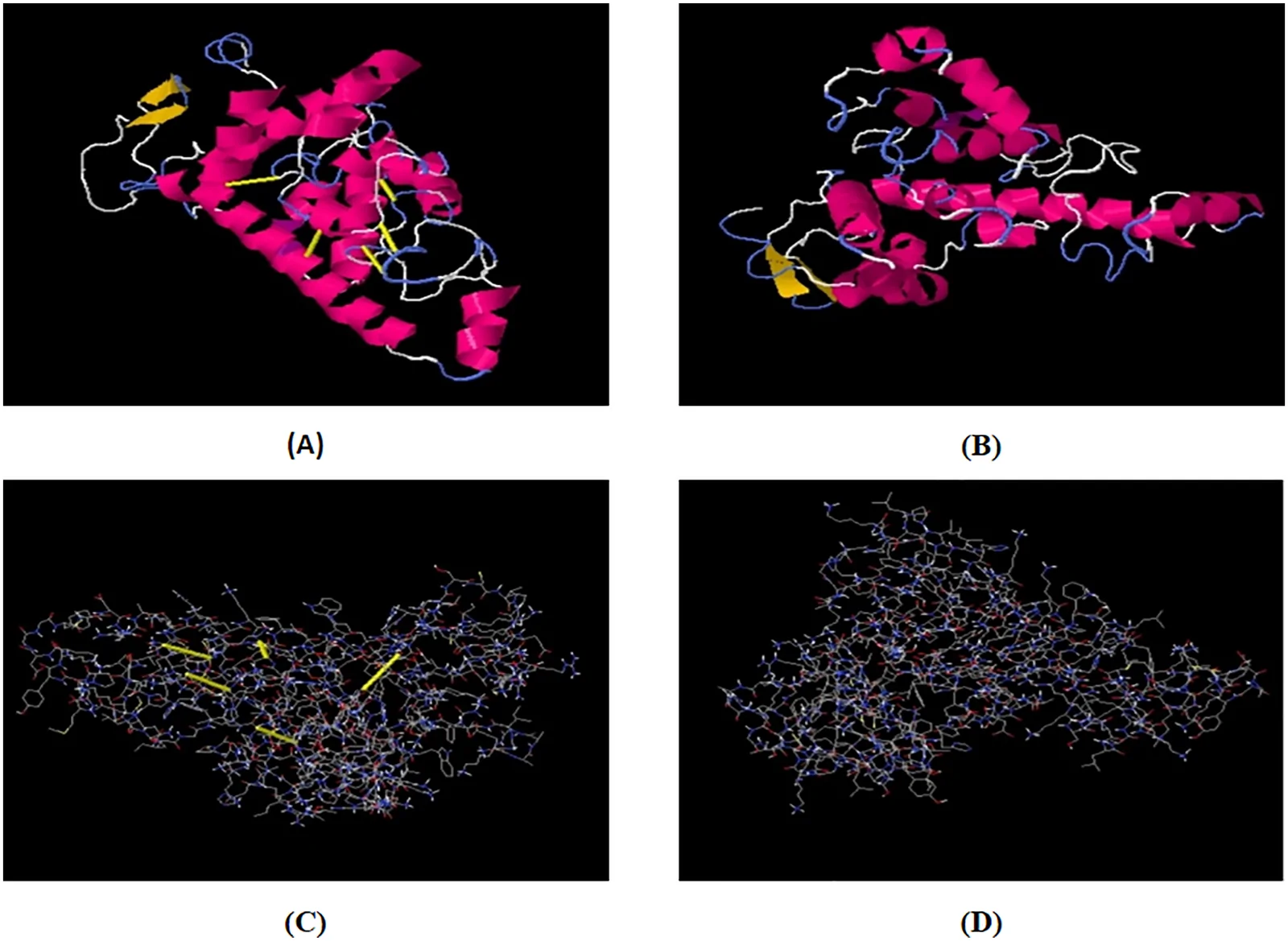
Disulfide engineering of vaccine construct V1. (A) Initial form (cartoon), (B) mutant form (cartoon), (C) initial form (wireframe), and (D) mutant form (wireframe). Yellow indicates hydrogen bonds. The web version of this article is recommended for interpretation of the color references in this figure legend.

### MD simulation of vaccine–receptor complex

A 200 ns MD simulation of the vaccine V1–TLR8 complex supported the structural stability of the docked complex under physiological conditions (Fig 10). RMSD trajectories showed rapid equilibration within approximately the first 20 ns, followed by stable convergence with average deviations of 0.25–0.45 nm, indicating limited structural drift. RMSF analysis revealed that flexibility was mainly restricted to terminal and loop regions (0.10–0.35 nm), whereas residues at the core interaction interface fluctuated below 0.20 nm. The complex maintained 40–60 intermolecular hydrogen bonds throughout the simulation, supporting persistent receptor binding. SASA values remained within 279–290 nm^2^, indicating limited changes in solvent exposure, and Rg values of 2.4–2.7 nm confirmed maintenance of overall compactness.

**Fig 10:**
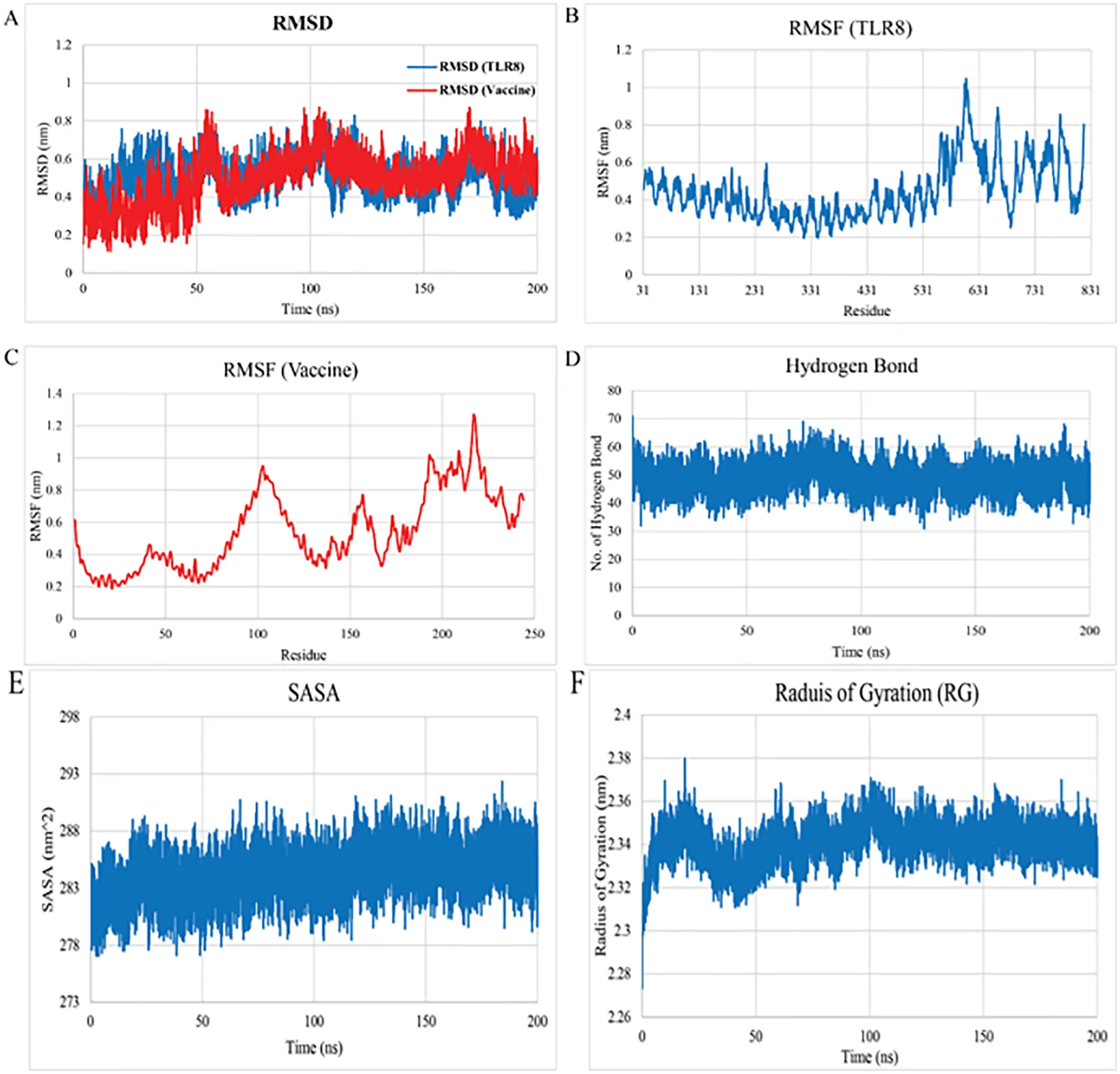
Molecular dynamics simulation of the vaccine V1–TLR8 complex. (A) RMSD trajectories of TLR8 and the vaccine construct. (B) RMSF profile of TLR8. (C) RMSF profile of the vaccine construct. (D) Intermolecular hydrogen-bond profile. (E) SASA trajectory. (F) Rg trajectory showing the compactness of the complex during simulation.

PCA indicated that PC1, PC2, and PC3 accounted for 28.37%, 9.84%, and 5.73% of total motion, respectively, with a cumulative contribution of 43.94% (Fig 11A). The first five eigenvectors explained 59.3% of conformational variance, whereas the first 20 eigenvectors accounted for 72.1%, suggesting that the dominant dynamics were governed by a limited number of collective motions. DCCM analysis showed strong positive correlations approaching +1.0 within residues 20–208 and 334–520, with anti-correlated motions approaching −1.0 between inter-domain regions around residues 208–334 (Fig 11B). These correlated and anti-correlated movement patterns indicate coordinated residue communication and support the dynamic stability of the V1–TLR8 complex.

**Fig 11:**
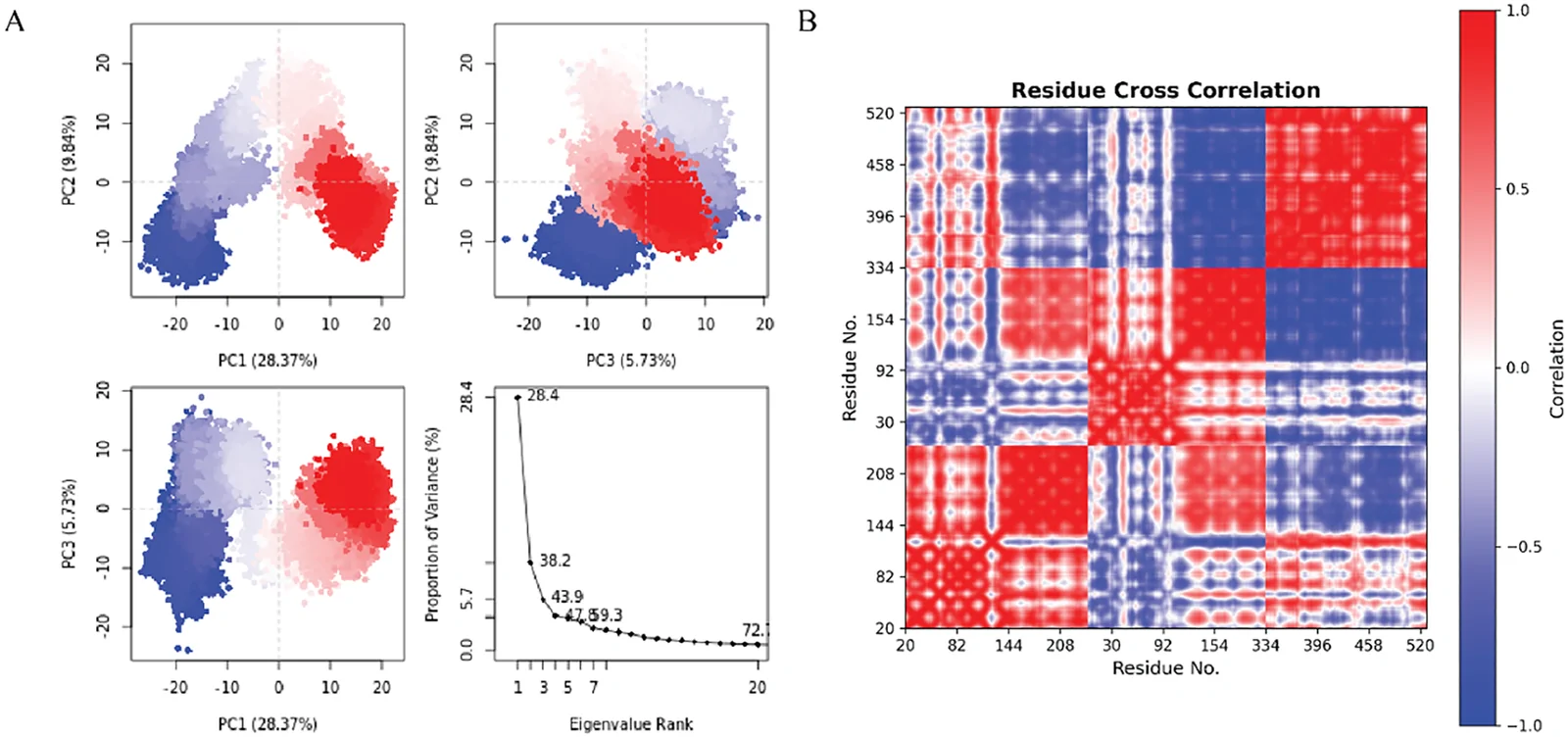
Principal component analysis (PCA) and dynamic cross-correlation matrix (DCCM) profiles of the vaccine–TLR8 complex. (A) PCA projections along PC1–PC2, PC2–PC3, and PC1–PC3 subspaces, with a scree plot showing variance explained by the first 20 eigenvectors. (B) DCCM heatmap illustrating correlated and anti-correlated residue motions; red indicates positive correlation, blue indicates negative correlation, and white indicates weak or near-zero correlation.

### MM/GBSA-based binding free energy analysis of the vaccine–TLR8 complex

MM/GBSA analysis confirmed favorable binding between vaccine construct V1 and TLR8, with a total binding free energy (ΔGbind) of −25.93 ± 3.49 kcal·mol ^−1^ (Table 4). Electrostatic interactions (ΔEelec = −70.22 ± 2.73 kcal·mol ^−1^) and van der Waals contributions (ΔEvdW = −43.73 ± 1.25 kcal·mol ^−1^) were the main favorable energetic components, producing a gas-phase free energy (ΔGgas) of −113.94 ± 3.25 kcal·mol ^−1^. Although solvation opposed binding (ΔGsolv = +88.02 ± 1.26 kcal·mol ^−^¹), the favorable nonbonded interactions were sufficient to maintain an overall negative ΔGbind. These results indicate that electrostatic and hydrophobic contacts contributed substantially to the stability of the V1–TLR8 complex, consistent with the MD trajectory behavior.

**Table 4:** Energy components contributing to the MM/GBSA binding profile of the vaccine V1–TLR8 complex.

Parameter	Vaccine V1–TLR8
van der Waals energy, ΔEvdW (kcal·mol ^−1^)	−43.73 ± 1.25
Electrostatic energy, ΔEelec (kcal·mol ^−1^)	−70.22 ± 2.73
Gas-phase free energy, ΔGgas (kcal·mol ^−1^)	−113.94 ± 3.25
Solvation free energy, ΔGsolv (kcal·mol ^−1^)	+88.02 ± 1.26
Total binding free energy, ΔGbind (kcal·mol ^−1^)	−25.93 ± 3.49

### Immune response simulation

C-ImmSim analysis predicted a coordinated immune response following antigen exposure (Fig 12). Antigen concentration increased immediately after administration and then declined as immune activation progressed. The antibody profile showed early IgM production followed by sustained IgG subclass responses, indicating maturation of humoral immunity. Helper T-cell and cytotoxic T-cell populations expanded substantially, reflecting activation of cellular immune mechanisms, whereas regulatory T-cell dynamics suggested controlled immune modulation. B-cell proliferation and differentiation into plasma cells supported sustained antibody production. Cytokine and interleukin profiles, including IFN-γ, IFN-β, IL-10, and IL-23, showed transient changes consistent with immune signaling and response coordination. Overall, the simulation supports the potential of V1 to induce durable humoral and cellular immunity.

**Fig 12:**
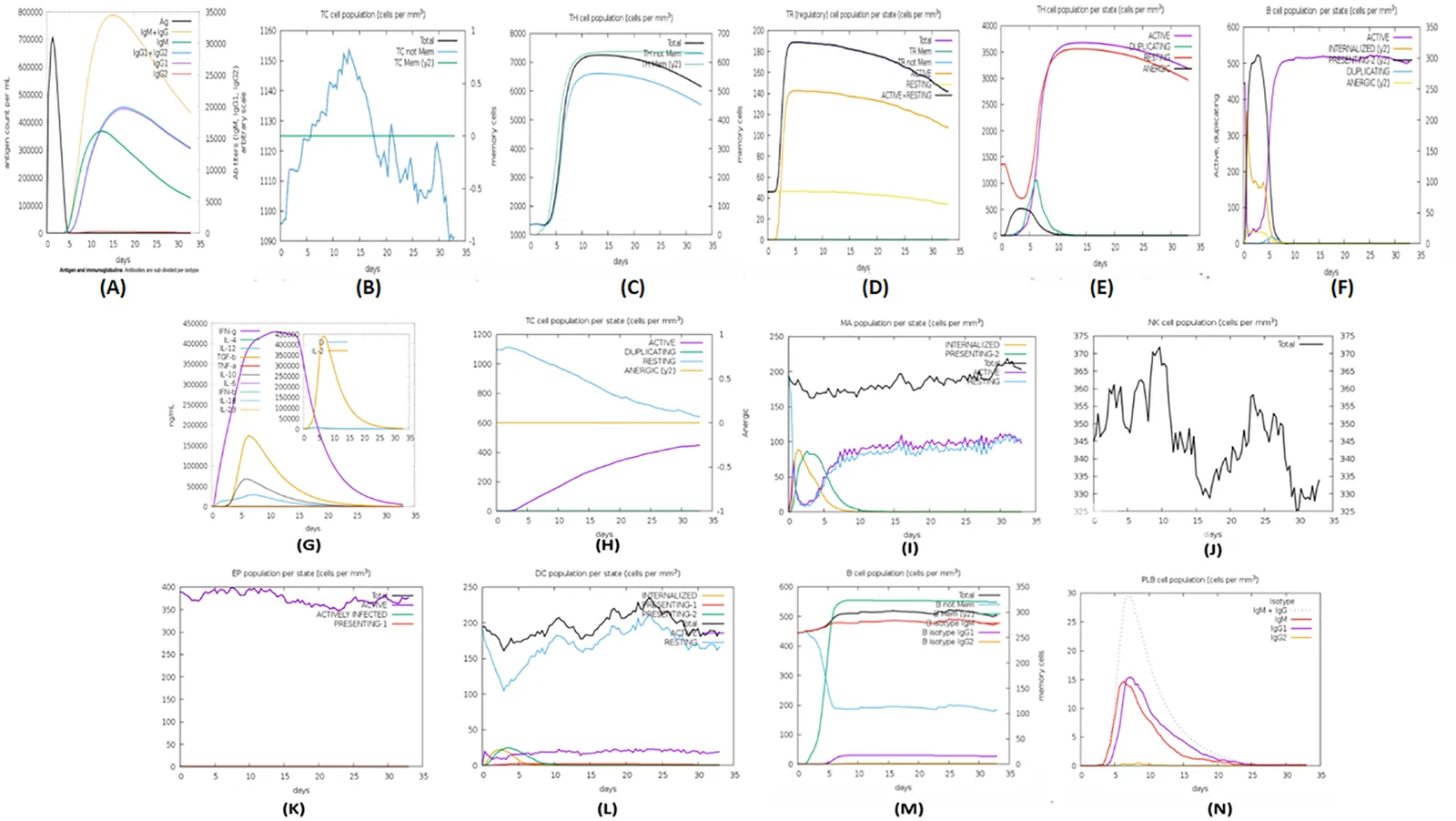
In silico immune simulation of the multi-epitope La Crosse virus vaccine construct. (A) Antigen and antibody response over time. (B) Total cytotoxic T-cell population dynamics. (C) Helper T-cell population dynamics. (D) Regulatory T-cell population by state. (E) Helper T-cell population by state. (F) B-cell population by state. (G) Cytokine concentration dynamics. (H) Cytotoxic T-cell population by functional state. (I) Macrophage population by state. (J) Natural killer cell population dynamics. (K) Epithelial cell population by state. (L) Dendritic cell population by state. (M) B-cell population and isotype distribution. (N) Antibody–antigen interaction or concentration curve.

### Disulfide bond simulation of vaccine candidate V1

Disulfide engineering of V1 using the DbD2 server identified 34 residue pairs with potential to form stabilizing disulfide linkages (S3 File). After applying energy (<2.5 kcal/mol) and χ3 angle (−87° to +97°) thresholds, five residue pairs—Thr89–Ala228, Ala91–Leu193, Pro106–Ala239, Tyr119–Gly174, and Ser192–Ala196—were selected for stabilization. ElliPro-based prediction of discontinuous B-lymphocyte epitopes identified eight conformational epitopes comprising 123 residues (S6 Table). These epitopes ranged from 3 to 46 amino acids in length and had scores between 0.687 and 0.768, indicating favorable conformational antigenicity.

### In silico cloning and RNA structure validation

Codon optimization of the V1 construct produced a CAI of 0.55 and a GC content of 68.03%, supporting compatibility with Escherichia coli expression. In silico cloning confirmed successful insertion of the optimized V1 coding sequence into the pET expression vector, yielding a recombinant plasmid of 4260 bp (Fig 13). RNA secondary-structure prediction generated a minimum free energy (MFE) structure of −342.82 kcal/mol. The MFE structure frequency within the ensemble was 0.00%, and the ensemble diversity was 207.22 (Fig 14). Most nucleotide positions exhibited low to moderate positional entropy, suggesting a generally well-defined structure, whereas higher entropy around positions 1000–1100 indicated localized flexibility or possible alternative conformations in that region.

**Fig 13:**
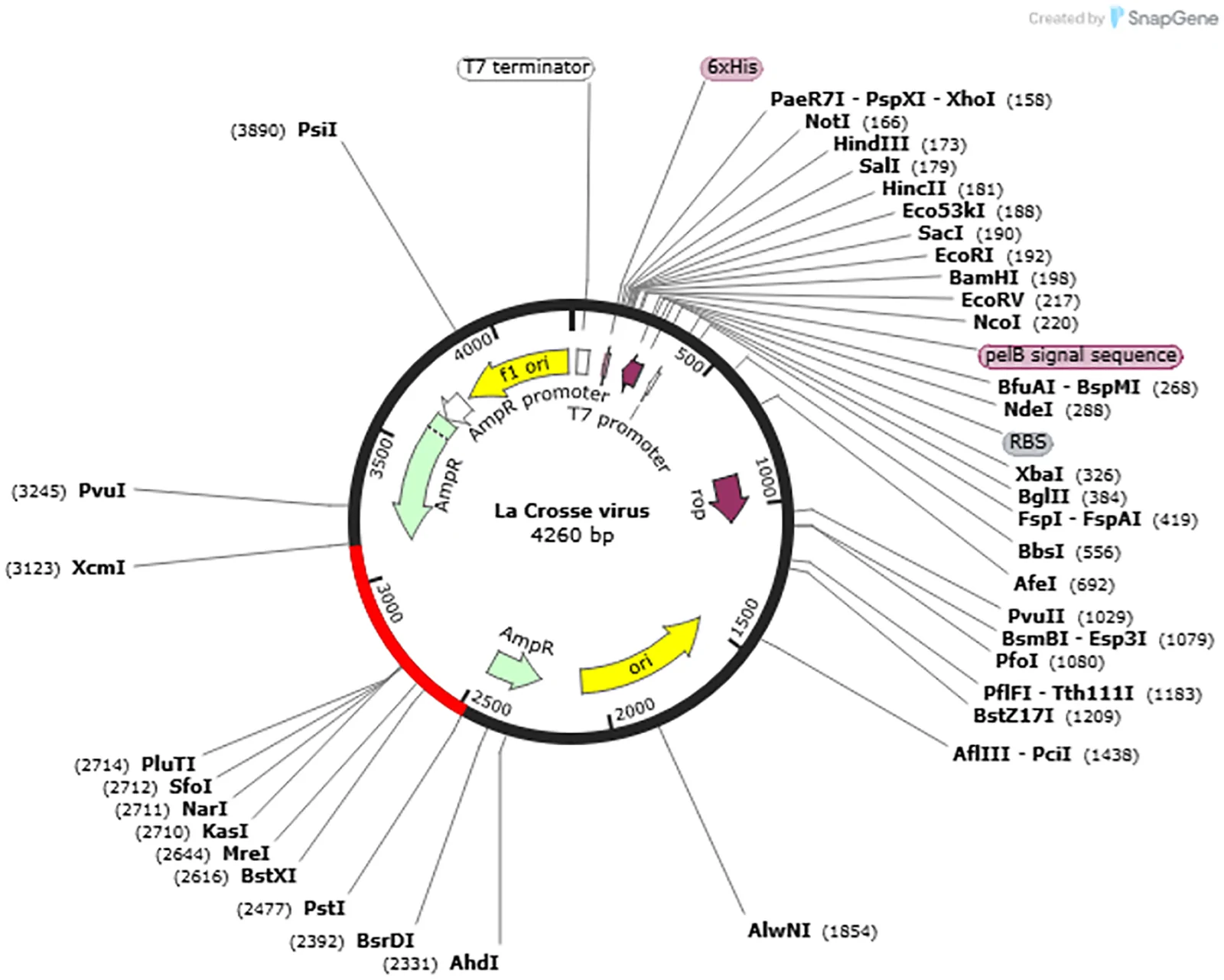
Circular map of the La Crosse virus vaccine construct. The inserted V1 construct is shown in red within the 4260 bp plasmid, with annotated open reading frames (ORFs) indicated by colored arrows. The web version of this article is recommended for interpretation of the color references in this figure legend.

**Fig 14:**
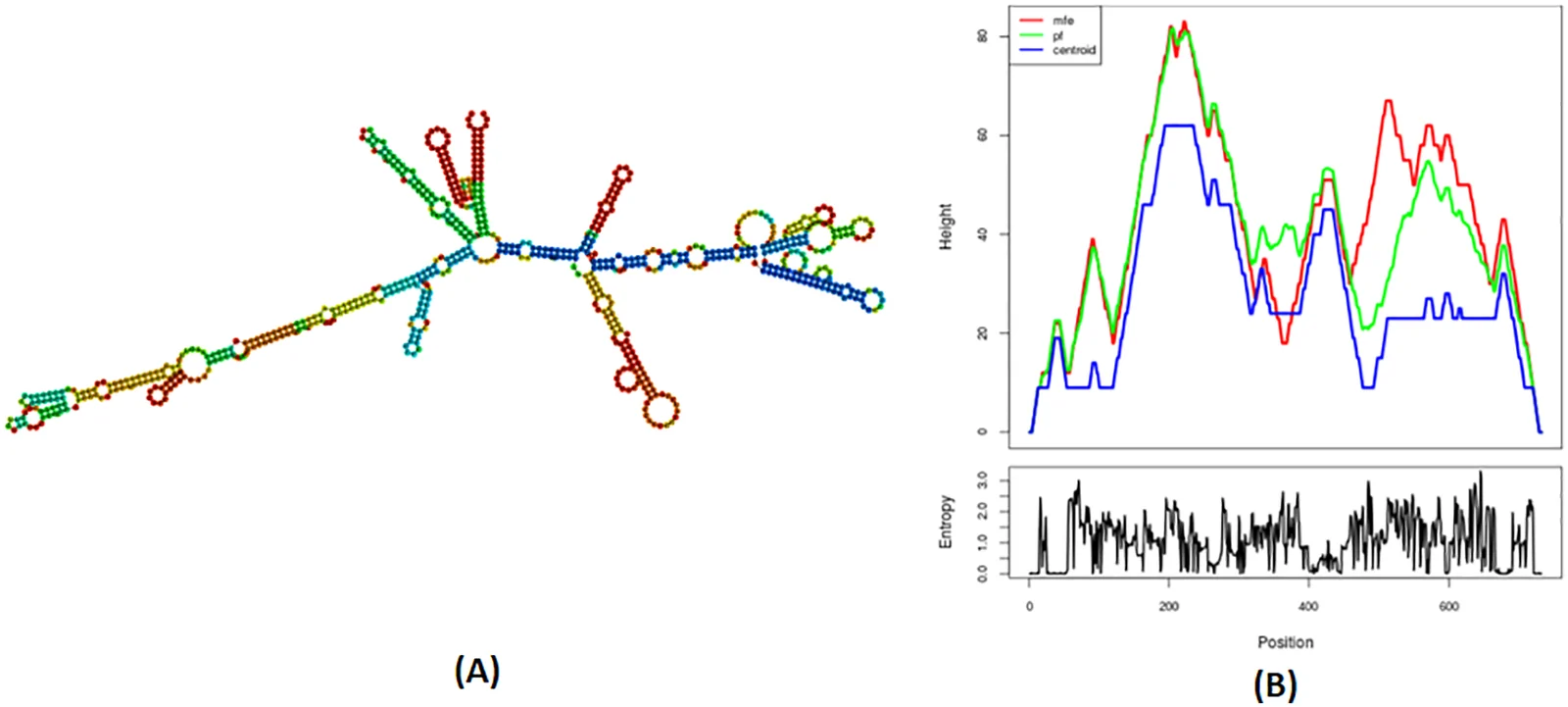
Predicted mRNA secondary structure of the developed vaccine construct V1. (A) Minimum free energy-based RNA secondary structure showing stem–loop formations. (B) Mountain plot comparing MFE (red), partition function (green), and centroid (blue) structures, with positional entropy indicating structural variability across the sequence.

## Discussion

This study presents a comprehensive immunoinformatics-based strategy for the rational design of a multi-epitope vaccine targeting La Crosse virus (LACV). The selection of the nucleoprotein as the primary antigen is supported by its conserved structure and essential role in viral replication, making it a suitable candidate for broad-spectrum vaccine development. Recent studies have demonstrated that structural proteins, including nucleoproteins, play a critical role in eliciting T-cell-mediated immune responses against orthobunyaviruses, further validating their suitability as vaccine targets [2,26].

The identification of conserved B-cell and T-cell epitopes represents a key strength of this study. Conserved epitopes are particularly important in combating rapidly evolving RNA viruses, as they reduce the likelihood of antigenic escape and ensure sustained vaccine efficacy across multiple strains. This approach aligns with recent findings emphasizing that epitope conservancy is a critical determinant of long-term vaccine effectiveness, particularly in arboviruses with expanding geographic distributions [1]. The high global population coverage observed in this study further supports the potential applicability of the vaccine across diverse HLA genotypes, addressing a major challenge in epitope-based vaccine design.

The incorporation of both cytotoxic T lymphocyte (CTL) and helper T lymphocyte (HTL) epitopes ensures the activation of comprehensive immune responses, including both cellular and humoral immunity. This dual activation is essential for viral clearance, as CD8 ⁺ T cells directly eliminate infected cells while CD4 ⁺ T cells facilitate antibody production and immune memory formation. Recent immunoinformatics studies have consistently demonstrated that multi-epitope vaccines integrating CTL, HTL, and B-cell epitopes exhibit superior immunogenicity compared to single-epitope or traditional subunit vaccines [7,9,27].

Among the designed constructs, V1 demonstrated superior antigenicity, solubility, and structural stability. These properties are critical determinants of vaccine efficacy, as they influence protein expression, folding, and interaction with immune receptors. Structural validation confirmed that the vaccine constructs possess favorable stereochemical properties, with the majority of residues located in energetically favorable regions. Such structural integrity is essential for maintaining epitope conformation and ensuring effective immune recognition [27].

Molecular docking analysis revealed strong binding interactions between the vaccine construct and Toll-like receptor 8 (TLR-8), suggesting a high potential for innate immune activation. TLR-8 is known to recognize viral RNA and plays a crucial role in initiating antiviral immune responses. The strong interaction observed in this study indicates that the vaccine construct may effectively trigger downstream signaling pathways, leading to cytokine production and adaptive immune activation. These findings are consistent with recent studies highlighting the importance of TLR engagement in enhancing vaccine-induced immunity [28,29].

The 200 ns MD simulation further substantiated the structural integrity and dynamic stability of the vaccine–TLR8 complex, as evidenced by stable RMSD and Rg values, low interface residue fluctuations, persistent hydrogen bonding, and conserved compactness. These findings suggest sustained receptor engagement and favorable conformational behavior required for effective innate immune activation. The PCA, DCCM, and MM/GBSA analyses collectively support the dynamic stability and favorable binding behavior of the vaccine–TLR8 complex. Similar observations have been associated with enhanced complex stability and immunogenic potential in recent computational vaccine studies [7,27,28,30,31].

The immune simulation analysis demonstrated robust activation of both humoral and cellular immune responses, including increased levels of IgM and IgG antibodies, T-cell proliferation, and cytokine production (e.g., IFN-γ and IL-10). These findings suggest the potential for long-lasting immunity and effective viral neutralization. Notably, the generation of memory B and T cells indicates the ability of the vaccine to provide sustained protection, which is a critical requirement for controlling viral infections [32,33]. Recent advances in computational immunology have shown that in silico immune simulations can reliably predict vaccine-induced immune responses, providing valuable insights prior to experimental validation (Khan et al., 2025).

Despite these promising findings, several limitations should be acknowledged. Firstly, the study relies entirely on computational predictions, which may not fully capture the complexity of biological systems. Factors such as post-translational modifications, protein folding dynamics in vivo, and host-specific immune variability may influence vaccine performance. Secondly, while the predicted epitopes demonstrated high antigenicity and non-allergenicity, experimental validation is required to confirm their immunogenicity and safety. Thirdly, the potential impact of viral evolution and mutation on epitope stability should be considered, particularly in the context of climate-driven expansion of mosquito vectors and changing transmission dynamics of LACV.

## Conclusion

This study successfully designed a multi-epitope vaccine candidate against La Crosse virus using immunoinformatics approaches. Among the constructs, V1 demonstrated superior antigenicity, structural stability, and strong interaction with immune receptors, supported by molecular dynamics and immune simulation analyses. The vaccine exhibited broad population coverage and favorable expression potential. These findings highlight the promise of computational vaccinology in accelerating vaccine development. However, experimental validation is required to confirm immunogenicity, safety testing in animal models, and eventually clinical trials to confirm the protective efficacy of the proposed vaccine construct.

### Artificial intelligence disclaimer

ChatGPT was used to correct grammar and improve the clarity of the manuscript text.

## Supporting information

S1 Table Accession information, geographic origin, and year of isolation of La Crosse virus nucleoprotein sequences used for multiple sequence alignment. (DOCX)

S2 Table ProtParam analysis of selected structural proteins. (DOCX)

S3 Table The anticipated B-cell epitopes allergenicity profile generated envelope polyprotein. (DOCX)

S4 Table Predicted T-cell epitopes (MHC-I peptides) of Nucleoprotein. (DOCX)

S5 Table Predicted T-cell epitopes (MHC-II peptides) of Nucleoprotein. (DOCX)

S6 Table Predicted Discontinuous Epitope(s) of vaccine Construct V1. (DOCX)

S1 File Predicted CTL (MHC-I) candidate epitopes. (XLSX)

S2 File Predicted HTL (MHC-II) candidate epitopes. (CSV)

S3 File Predicted disulfide residue pairs. (CSV)

S4 File Supplimentary Figures. S1 Fig. Predicted secondary structural organization of three viral proteins for vaccine V1 (A-B), V2 (C-D) and V3 (E-F), highlighting their conformational stability and functional motifs. S2 Fig. Tertiary structure prediction and validation of vaccine for construct V2 (A) The ERRAT value of vaccine was calculated to assess model quality. (B) Verify3D shows a score for our selected vaccine mode (blue colour determines the average score), (C) Ramachandran plot analysis; and for candidate V3 (D) The ERRAT value of vaccine was calculated to assess model quality. (E) Verify3D shows a score for our selected vaccine mode (blue colour determines the average score), (F) Ramachandran plot analysis. S3 Fig. Conformational B-cell epitopes have been identified inside the vaccine design, with light-yellow spheres denoting epitopes of differing residue lengths and binding affinities. (A) THR 89- ALA 228 with 0.768; (B) ALA 91- LEU 193 with 0.756; (C) PRO 106- ALA 239 with 0.748; (D) TYR 119- GLY 174 with 0.702; (E) SER 192 – ALA 196 with 0.687. For interpretation of the references to color in this figure legend, the reader is referred to the web version of this article.
(PPTX)
