# Supplementary material for: Designing a multi-epitope vaccine against La Crosse virus using immunoinformatics approach
Source: PLoS One. 2026 Oct 13;21(10):e0359567. doi: 10.1371/journal.pone.0359567 (PMC13660988; doi:10.1371/journal.pone.0359567)
Supplement: Supplementary file 1 [file pone.0359567.s001.docx]

**Table S1.** Accession information, geographic origin, and year of isolation of La Crosse virus nucleoprotein sequences used for multiple sequence alignment.

| Accession ID | Country | Years of isolation |
| --- | --- | --- |
| YP_010839408.1 | USA | 2023 |
| 4BHH_B | USA | 2023 |
| AKP04430.1 | USA | 2015 |
| WHP37885.1 | USA | 2023 |
| ADH04725.1 | USA | 2011 |
| NP_671970.1 | USA | 2018 |
| 4BGP_A | USA | 2020 |
| ADG01819.1 | USA | 2010 |
| ADG01817.1 | USA | 2010 |
| ADD85203.1 | USA | 2010 |
| ADD85209.1 | USA | 2010 |
| AAB81525.1 | USA | 2016 |
| AAB81524.1 | USA | 2016 |
