# Supplementary material for: Designing a multi-epitope vaccine against La Crosse virus using immunoinformatics approach
Source: PLoS One. 2026 Oct 13;21(10):e0359567. doi: 10.1371/journal.pone.0359567 (PMC13660988; doi:10.1371/journal.pone.0359567)
Supplement: Supplementary file 2 [file pone.0359567.s002.docx]

**Table S2. ProtParam analysis of selected structural proteins**.

| **Protein** | **Accession ID** | **Molecular Weight** | **Instability Index** | **Half-life** | **Theoretical pI** | **No. of Amino acids** | **Total No. of Atoms** | **Estinction Co-efficient** | **VaxiJen score** |  |
| --- | --- | --- | --- | --- | --- | --- | --- | --- | --- | --- |
| **Nucleoprotein** | P04873 | 26530.25 | 29.22 | 30 hours | 8.82 | 235 | 3722 | 39420 | 0.4325 | |
