# Supplementary material for: Designing a multi-epitope vaccine against La Crosse virus using immunoinformatics approach
Source: PLoS One. 2026 Oct 13;21(10):e0359567. doi: 10.1371/journal.pone.0359567 (PMC13660988; doi:10.1371/journal.pone.0359567)
Supplement: Supplementary file 3 [file pone.0359567.s003.docx]

**Supplementary Table S3. The anticipated B-cell epitopes allergenicity profile generated envelope polyprotein**

| **Protein** | **Start** | **End** | Length | **Peptide** | **Vaxijen score** | **Allergenicity** | **Final Assessment** |
| --- | --- | --- | --- | --- | --- | --- | --- |
| **Nucleoprotein** | 33 | 39 | 07 | SLNLAAV | **1.4974** | NON-ALLERGEN | **Selected** |
|  | 170 | 177 | 08 | QGMMDPQY | **1.2862** | NON-ALLERGEN | **Selected** |
|  | 20 | 25 | 06 | DAGYMD | **1.2701** | NON-ALLERGEN | **Selected** |
|  | 31 | 37 | 07 | AESLNLA | **1.1855** | NON-ALLERGEN | **Selected** |
