# Supplementary material for: Designing a multi-epitope vaccine against La Crosse virus using immunoinformatics approach
Source: PLoS One. 2026 Oct 13;21(10):e0359567. doi: 10.1371/journal.pone.0359567 (PMC13660988; doi:10.1371/journal.pone.0359567)
Supplement: Supplementary file 4 [file pone.0359567.s004.docx]

**Table S4. Predicted T-cell epitopes (MHC-I peptides) of Nucleoprotein**

| **Start** | **End** | **Length** | **Peptide** | **HLA** | **Vaxijen Score** | **Antigen or Non-Antigen** | **Cell I Immunigenesity** | **Topology** | **AllerTOP v. 2.0** | **AllerCatPro 2.0** | **Toxinpred** | **Final Assessment** |
| --- | --- | --- | --- | --- | --- | --- | --- | --- | --- | --- | --- | --- |
| 05 | 13 | 09 | ASTGANGFD | HLA-A*30:02 | 0.6017 | Antigen | 0.17571 | Outsi-de | NON-ALLERGEN | NON-ALLERGEN | Non-Toxin |  |
| **05** | **14** | **10** | **ASTGANGFDP** | **HLA-B*57:01** | **0.7955** | **Antigen** | **0.22216** | **Outsi-de** | **NON-ALLERGEN** | **NON-ALLERGEN** | **Non-Toxin** |  |
| 06 | 14 | 09 | EIYLSFFPG | HLA-A*33:01 | 0.5181 | Antigen | 0.02906 | Outsi-de | NON-ALLERGEN | NON-ALLERGEN | Non-Toxin |  |
| 06 | 15 | 10 | EIYLSFFPGT | HLA-A*68:02 | 0.6271 | Antigen | 0.06118 | Outsi-de | NON-ALLERGEN | NON-ALLERGEN | Non-Toxin |  |
| 18 | 27 | 10 | FLETFKFYPL | HLA-A*02:01 | 0.5798 | Antigen | 0.07616 | Outsi-de | NON-ALLERGEN | NON-ALLERGEN | Non-Toxin |  |
| 01 | 10 | 10 | FYDVASTGAN | HLA-A*23:01 | 0.7501 | Antigen | 0.01374 | Outsi-de | NON-ALLERGEN | NON-ALLERGEN | Non-Toxin |  |
| 07 | 15 | 09 | IYLSFFPGT | HLA-A*30:01 | 0.7505 | Antigen | 0.06457 | Outsi-de | NON-ALLERGEN | NON-ALLERGEN | Non-Toxin |  |
| 07 | 16 | 10 | IYLSFFPGTE | HLA-A*23:01 | 0.7523 | Antigen | 0.09877 | Outsi-de | NON-ALLERGEN | NON-ALLERGEN | Non-Toxin |  |
| **23** | **32** | **10** | **KFYPLTIGIH** | **HLA-A*30:01** | **1.8333** | **Antigen** | **0.24628** | **Outsi-de** | **NON-ALLERGEN** | **NON-ALLERGEN** | **Non-Toxin** |  |
| 03 | 11 | 09 | LAAVRIFFL | HLA-B*51:01 | 0.4951 | Antigen | 0.39712 | Outsi-de | NON-ALLERGEN | NON-ALLERGEN | Non-Toxin |  |
| 03 | 12 | 10 | LAAVRIFFLN | HLA-B*58:01 | 0.4110 | Antigen | 0.43676 | Outsi-de | NON-ALLERGEN | NON-ALLERGEN | Non-Toxin |  |
| 19 | 27 | 09 | LETFKFYPL | HLA-B*40:01 | 0.6800 | Antigen | 0.021 | Outsi-de | NON-ALLERGEN | NON-ALLERGEN | Non-Toxin |  |
| 01 | 10 | 10 | LNLAAVRIFF | HLA-A*23:01 | 0.4343 | Antigen | 0.34351 | Outsi-de | NON-ALLERGEN | NON-ALLERGEN | Non-Toxin |  |
| 09 | 17 | 09 | LSFFPGTEM | HLA-B*15:01 | 0.7392 | Antigen | 0.26816 | Outsi-de | NON-ALLERGEN | NON-ALLERGEN | Non-Toxin |  |
| 02 | 11 | 10 | NLAAVRIFFL | HLA-A*02:01 | 0.4751 | Antigen | 0.43515 | Outsi-de | NON-ALLERGEN | NON-ALLERGEN | Non-Toxin |  |
| 06 | 15 | 10 | STGANGFDPD | HLA-A*68:02 | 0.4821 | Antigen | 0.19951 | Outsi-de | NON-ALLERGEN | NON-ALLERGEN | Non-Toxin |  |
| 07 | 15 | 09 | TGANGFDPD | HLA-B*35:01 | 0.5084 | Antigen | 0.16163 | Outsi-de | NON-ALLERGEN | NON-ALLERGEN | Non-Toxin |  |
| **08** | **16** | **09** | **YLSFFPGTE** | **HLA-A*02:03** | **0.7558** | **Antigen** | **0.21894** | **Outsi-de** | **NON-ALLERGEN** | **NON-ALLERGEN** | **Non-Toxin** |  |
