# Supplementary material for: Designing a multi-epitope vaccine against La Crosse virus using immunoinformatics approach
Source: PLoS One. 2026 Oct 13;21(10):e0359567. doi: 10.1371/journal.pone.0359567 (PMC13660988; doi:10.1371/journal.pone.0359567)
Supplement: Supplementary file 5 [file pone.0359567.s005.docx]

**Table S5. Predicted T-cell epitopes (MHC-II peptides) of Nucleoprotein**

| **Start** | **End** | **Length** | **Peptide** | **HLA** | **Vaxijen Score** | **Antigen or Non-Antigen** | **Cell I Immunigenesity** | **Topology** | **AllerTOP v. 2.0** | **AllerCatPro 2.0** | **Toxinpred** | **Final Assesment** |
| --- | --- | --- | --- | --- | --- | --- | --- | --- | --- | --- | --- | --- |
| **22** | **36** | **15** | **FKFYPLTIGIHRVKQ** | **HLA-DPA1*02:01/DPB1*14:01** | **1.4319** | **Antigen** | **0.33174** | **Outside** | **NON-ALLERGEN** | **NON-ALLERGEN** | **Non-Toxin** |  |
| 01 | 15 | 15 | FYDVASTGANGFDPD | HLA-DRB1*07:01 | 0.5966 | Antigen | 0.18378 | Outside | NON-ALLERGEN | NON-ALLERGEN | Non-Toxin |  |
| **23** | **37** | **15** | **KFYPLTIGIHRVKQG** | **HLA-DRB1*11:01** | **1.3465** | **Antigen** | **0.17792** | **Outside** | **NON-ALLERGEN** | **NON-ALLERGEN** | **Non-Toxin** |  |
| 03 | 17 | 15 | LAAVRIFFLNAAKAK | HLA-DRB3*02:02 | 0.4735 | Antigen | 0.36093 | Outside | NON-ALLERGEN | NON-ALLERGEN | Non-Toxin |  |
| **19** | **33** | **15** | **LETFKFYPLTIGIHR** | **HLA-DPA1*01:03/DPB1*04:01** | **0.8295** | **Antigen** | **0.33972** | **Outside** | **NON-ALLERGEN** | **NON-ALLERGEN** | **Non-Toxin** |  |
| 01 | 15 | 15 | LNLAAVRIFFLNAAK | HLA-DPA1*02:01/DPB1*05:01 | 0.4996 | Antigen | 0.56106 | Outside | NON-ALLERGEN | NON-ALLERGEN | Non-Toxin |  |
| **21** | **35** | **15** | **TFKFYPLTIGIHRVK** | **HLA-DPA1*02:01/DPB1*14:01** | **1.4458** | **Antigen** | **0.45085** | **Outside** | **NON-ALLERGEN** | **NON-ALLERGEN** | **Non-Toxin** |  |

**Supplementary Table S6. Predicted Discontinuous Epitope(s) of vaccine Construct V1:**

| **No.** | **Residues** | **Number of residues** | **Score** |
| --- | --- | --- | --- |
| 1 | A:Y75, A:P76, A:I79 | 3 | 0.768 |
| 2 | A:A66, A:A67, A:A68, A:G69, A:G70, A:G71, A:S72, A:K73, A:F74 | 9 | 0.756 |
| 3 | A:P131, A:K190, A:S192, A:L193, A:N194, A:L195, A:A196, A:A197, A:V198, A:K199, A:K200, A:G202, A:M203, A:M204, A:D205, A:P206, A:Q207, A:Y208, A:K209, A:K210, A:D211, A:A212, A:G213, A:Y214, A:M215, A:D216, A:K217, A:K218, A:A219, A:E220, A:S221, A:L222, A:N223, A:K226 | 34 | 0.748 |
| 4 | A:E1, A:A2, A:A3, A:A4, A:K5, A:G6, A:I7, A:I8, A:N9, A:T10, A:L11, A:Q12, A:Y15, A:R19, A:R22, A:A24, A:V25, A:L26, A:S27, A:C28, A:L29, A:P30, A:K31, A:E32, A:E33, A:Q34, A:I35, A:G36, A:K37, A:C38, A:S39, A:T40, A:R41, A:G42, A:R43, A:K44, A:C45, A:C46, A:R47, A:R48, A:K49, A:E51, A:G241, A:G242, A:G243, A:S244 | 46 | 0.702 |
| 5 | A:G112, A:P113, A:G114, A:F118, A:P139, A:I142, A:G143, A:I144, A:H145, A:R146, A:V147, A:K148, A:Q149, A:G150, A:P151, A:G152, A:P153, A:G154, A:K155, A:F156, A:Y157, A:P158, A:L159, A:T160, A:I161, A:G162, A:I163, A:H164, A:R165, A:K167, A:Q168 | 31 | 0.687 |
